# Metabolic syndrome and risk of incident all-cause dementia, Alzheimer’s disease and vascular dementia: a systematic review and meta-analysis of longitudinal studies

**DOI:** 10.1186/s13195-025-01825-4

**Published:** 2025-08-23

**Authors:** Danial Qureshi, Naomi E. Allen, Elżbieta Kuźma, Thomas J. Littlejohns

**Affiliations:** 1 Nuffield Department of Population Health, University of Oxford, Oxford, OX3 7LF UK; 2 UK Biobank Ltd, Stockport, SK3 0SA UK; 3 Faculty of Medicine, Albertinen Krankenhaus/Albertinen Haus gGmbH, Academic Teaching Hospital, University of Hamburg, 22459 Hamburg, Germany

**Keywords:** Metabolic syndrome, Dementia, Alzheimer’s disease, Vascular dementia, Systematic review, Meta-analysis, Longitudinal studies

## Abstract

**Background:**

Metabolic syndrome (MetS) comprises several co-occurring vascular and cardiometabolic characteristics and might represent a novel modifiable risk factor for dementia, though findings remain inconsistent. To clarify this, we conducted a systematic review and meta-analysis of longitudinal studies investigating the association between MetS with risk of incident all-cause dementia, Alzheimer’s disease and vascular dementia.

**Methods:**

We searched Medline, Embase and PsycINFO databases (from inception to Feb 19th, 2024) for longitudinal cohort studies investigating the association of MetS with incident all-cause dementia or key subtypes, including Alzheimer’s, vascular, Lewy Body or other dementias. Random-effects models were used to estimate pooled hazard ratios (pHR) and 95% confidence intervals (CI). Risk of bias was assessed using the Quality Assessment Tool for Quantitative Studies.

**Results:**

Of 4,719 studies identified, fourteen studies met the eligibility criteria. These studies combined included 4,345,741 participants who were initially free of dementia. Pooled estimates showed that, compared to participants with no MetS, those with MetS had a significantly greater risk of incident all-cause dementia (4,307,830 participants, 27,708 cases, pHR: 1.12, 95% CI: 1.08–1.15, *I*^*2*^ = 12.0%). No significant association was observed for incident Alzheimer’s disease (4,109,436 participants, 14,890 cases, pHR: 0.91, 95% CI: 0.72–1.15, *I*^2^: 41.0%) or vascular dementia (4,109,436 participants, 2,642 cases, pHR: 1.40, 95% CI: 0.96–2.06, *I*^2^: 59.0%). Associations remained similar in subgroup analyses restricted to studies reporting results for those aged 65 + years: all-cause dementia (pHR: 1.08, 95% CI: 1.00-1.16, *I*^2^: 15.0%), Alzheimer’s disease (pHR: 0.85, 95% CI: 0.65–1.11, *I*^2^: 0.0%), and vascular dementia (pHR: 1.55, 95% CI: 0.71–3.39, *I*^2^: 75.0%).

**Conclusions:**

Our findings demonstrate that MetS may be an important risk factor for developing dementia, and represent a potential target for prevention. Further studies are needed to understand the influence of MetS on specific dementia subtypes.

**Supplementary Information:**

The online version contains supplementary material available at 10.1186/s13195-025-01825-4.

## Background

Dementia is a leading cause of morbidity and mortality worldwide and reducing the burden through risk reduction strategies represents an important public health goal [1]. Several modifiable cardiometabolic and vascular risk factors for dementia have been identified as key targets for dementia prevention, including diabetes, obesity, hypertension, and hyperlipidaemia [1]. Previous research has generally focused on the individual contribution of each of these factors to dementia risk. However, these factors often co-occur in individuals, commonly referred to as metabolic syndrome (MetS) [2]. Generally, the presence of at least three out of the five characteristics constitutes a diagnosis of MetS: (1) a large waist circumference; (2) high triglycerides; (3) high blood pressure; (4) high blood glucose; and (5) reduced high-density lipoprotein (HDL) cholesterol [2]. MetS affects approximately 20–25% of adults worldwide, and its role as a potential risk factor for dementia is gaining increasing interest [2]. 

A 2019 meta-analysis of six longitudinal studies found no association between MetS and incident all-cause dementia (pHR, 1.12, 95% CI: 0.94–1.33) [3]and a 2021 meta-analysis of four longitudinal studies found no association between MetS and Alzheimer’s disease (pHR, 0.80, 95% CI: 0.61–1.05) [4]. However, all but one of the studies that contributed to these reviews were published more than ten years ago and had sample sizes ranging from a few hundred to a few thousand participants. Consequently, the statistical precision of the effect estimates and ability to conduct important subgroup analyses was limited in these previous meta-analyses. Furthermore, the majority of studies had short follow-up periods, for example, the mean follow-up of the studies included in the 2021 meta-analysis was between 3.5 and 4.4 years. However, dementia pathology develops many years prior to a clinical diagnosis and there is a strong risk of reverse causation bias in studies with short-follow-up periods. Recently, various large-scale longitudinal studies with long-term follow-up have been conducted which may help to clarify whether MetS is a risk factor for dementia, and thus, a further comprehensive investigation of the existing literature is warranted.

We conducted an updated systematic review and meta-analysis of longitudinal studies investigating the associations between MetS and incident dementia, including dementia subtypes. We also investigated whether key study or population characteristics, such as age, sex, ethnicity and genetic predisposition for dementia modify the association, and conducted additional sensitivity and exploratory analyses to assess the robustness of findings and further potential sources of heterogeneity.

## Methods

This systematic review was conducted according to the Preferred Reporting Items for Systematic Reviews and Meta-Analyses (PRISMA) guidelines [5] and registered with the International Prospective Register of Systematic Reviews (PROSPERO); Registration Number: CRD42024549846.

### Search strategy, study selection and data extraction

Following a pre-defined protocol (see Methods S1), searches were developed for Medline, Embase, and PsycINFO via OvidSP. Subject headings and free-text terms were included, and all searches were peer-reviewed by an experienced librarian prior to execution. Full search strategy details are available in Tables S1-S3. The initial searches were conducted on February 9th, 2022, and subsequently updated on February 19th, 2024. Searches were supplemented by backward and forward citation searches of included studies via Web of Science. Additionally, automated e-mail alerts were set up to identify studies published after the updated search date.

This systematic review was restricted to longitudinal studies (in English, among adults ≥ 18 years) investigating the association between MetS (assessed at recruitment) and risk of incident all-cause dementia or key dementia subtypes, which included Alzheimer’s disease, vascular dementia, Lewy body dementia, and frontotemporal dementia. The comparison group was no prevalent MetS. The inclusion and exclusion criteria for the exposure of MetS were defined broadly to encompass all possible established standardised definitions used for MetS diagnoses in adults. Two reviewers (DQ & EK) independently screened titles and abstracts based on the inclusion and exclusion criteria and independently reviewed the full-text articles for potentially relevant studies. Any discrepancies were resolved by discussion, if necessary, with a third reviewer (TJL).

Study characteristics and fully adjusted results were extracted by one reviewer (DQ) and checked by a second reviewer (EK). Corresponding authors of eleven studies were contacted for clarification or additional data that was not reported [6–14] of whom four responded [10, 14–16].

### Assessment of risk of bias

Two reviewers (DQ, TJL) independently assessed the risk of bias using the Effective Public Health Practice Project Quality Assessment Tool for Quantitative Studies [17]. Any discrepancies were resolved by discussion with a third reviewer (EK). Overall risk of bias and potential sources of bias (i.e., selection bias, study design, confounders, blinding, data collection, withdrawals and drop-outs) were rated as ‘strong’, ‘moderate’, or ‘weak’ according the tool dictionary (Methods S1) [17]. Studies with ‘0’, ‘1’ or ‘≥2’ weak component ratings received an overall rating of ‘strong’, ‘moderate’ or ‘weak’, respectively.

### Data analysis

Included studies were categorised based on the main outcomes examined, which included: (1) incident all-cause dementia; (2) incident Alzheimer’s disease; and (3) incident vascular dementia. Given the heterogeneity across study characteristics, the data was pooled using random effects meta-analysis models [18] as pre-specified in the systematic review protocol (Methods S1, Additional File). Statistical heterogeneity was assessed using Cochran’s Chi-squared test (Chi^2^); *p* < 0.05 indicated significant heterogeneity) and the I-squared statistic (*I*^2^); a value of < 25%, 25–75% and > 75% were classified as low, moderate and high heterogeneity, respectively) [19]. Where studies reported multiple models, only estimates from the fully-adjusted model were included in the meta-analysis. For consistency and appropriate comparison, meta-analyses were performed using only studies reporting hazard ratios (HR) and 95% confidence interval (CI) values, and only studies that adopted similar definitions to define the main exposure (i.e., binary MetS variable). Those adopting exposure definitions that were not comparable to other studies were therefore excluded from the meta-analyses and synthesised narratively. Subgroup meta-analyses were performed to examine the association of MetS and dementia by: (1) the age at recruitment; (2) the MetS criteria adopted; (3) ethnicity; and (4) risk of bias ratings.

In addition, several exploratory and sensitivity analyses were conducted to assess the robustness of findings and explore potential sources of heterogeneity: (1) to address potential for misclassification of dementia diagnoses, we excluded studies that relied solely on passively collected data to define dementia diagnoses (e.g., health record linkage without clinical adjudication); (2) to account for the potential mediating role of vascular factors in the association between MetS and vascular dementia, we excluded studies that adjusted for intermediate outcomes such as stroke and cardiovascular disease; (3) to explore the influence of regional variation in MetS prevalence, we repeated the main meta-analysis excluding studies conducted in the United States; (4) to evaluate the impact of prolonged or persistent MetS on dementia risk, we analysed studies reporting longitudinal changes in MetS status over time; and (5) to assess potential dose-response relationships, we conducted an analysis of studies reporting dementia risk by the number of MetS components present.

Univariate meta-regression models were used to investigate the effects of potential moderators in the relationship between MetS and dementia risk for outcomes with at least ten studies included in a meta-analysis [20]. Meta-regression models were fit by regressing pooled HRs of dementia risk on the following factors: (1) publication year; (2) mean/median follow-up duration (in years); (3) mean/median age at baseline (in years); (4) use of MetS criteria: National Cholesterol Education Program - Adult Treatment Panel III (NCEP-ATP III; yes vs. no); (5) proportion of female participants (%); (6) adjustment for Apolipoprotein-E (*APOE*)-ε4 carrier status (yes vs. no); (7) ethnicity of the study cohort (predominantly White vs. predominantly non-white); and (8) geographic region of the study cohort (Western vs. non-Western). Where both mean and median values were reported for the variables of age or follow-up duration, the mean was used; if the mean was unavailable, the median was used. An adjusted p-value was calculated based on the permutation test to account for multiple testing [21–23]. 

Publication bias was assessed for all outcomes using funnel plots, and Egger’s statistic was calculated for outcomes with at least 10 studies included in the meta-analysis [24, 25]. All statistical analyses presented in this study were conducted in R version 4.2.2 (RStudio version 2023.12.1 + 402). Meta-analyses were performed using the “metagen” function and meta-regression models were fit using the “metareg” function, both of which are available in the “meta” package [26]. 

## Results

### Included studies

**Fig. 1:**
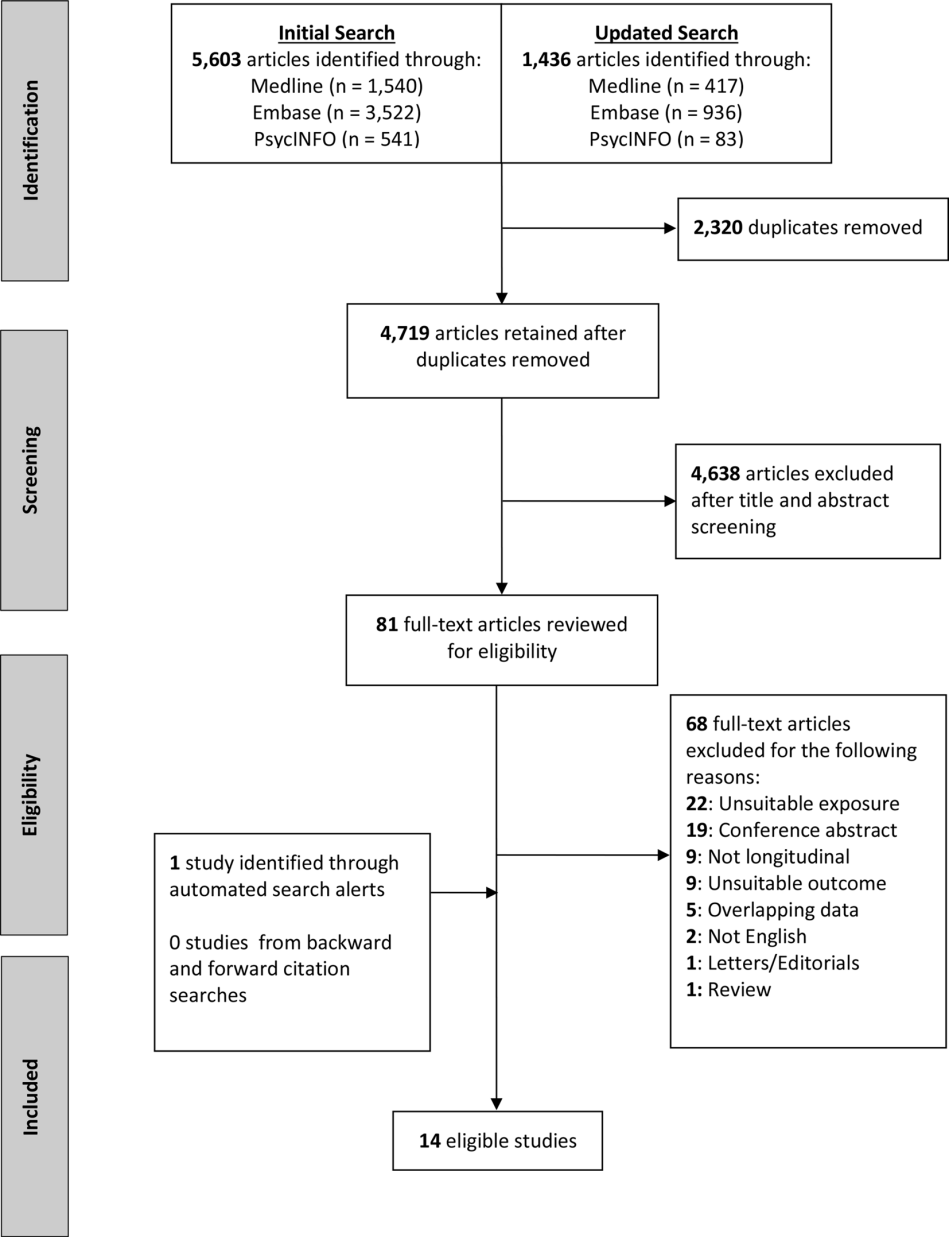
Flow chart of study selection and exclusion

The final literature search resulted in 7,903 publications. After removing 2,320 duplicates, 4,638 articles were excluded based on title and abstract screening, resulting in 81 publications for full-text review. A total of 14 studies met the inclusion criteria for the systematic review, which included a total of 4,345,741 participants who were initially free of dementia (see Fig. 1 for flow chart).

Of the 14 included studies, eight were from Europe [6, 8, 12–14, 16, 27, 28]four from Asia [7, 9, 11, 29]one from the United States [10]and one covered populations from both Australia and the United States (Table 1) [15]. Twelve studies used data from research cohorts [6–9, 11–16, 27–29], while two used data from medical records cohorts [9, 10]. The most commonly adopted definition for ascertaining MetS was the NCEP-ATP III criteria (eight studies) [6–8, 10, 12–14, 29]. MetS prevalence ranged from 15.8% 12 to 55% 10 across studies. All-cause dementia or its key subtypes were ascertained using a combination of: medical records, anti-dementia prescription drug use, or adjudication following established criteria for dementia, neuropsychological evaluations, neurological exams and/or brain magnetic resonance imaging (MRI). Analytic sample sizes varied between the studies, ranging from 59 participants in a memory clinic cohort study (the Netherlands) [6] to 4,106,590 participants from the largest medical records cohort study (South Korea) [9]. 

**Table 1:** Characteristics of all included studies

Study author, publication year	Region (data source)	Ethnicity	Study design	Setting	Analytic sample size	% Female	Mean baseline age in years (SD)	Mean follow-up length in years (SD)	Exposure: MetS criteria	Outcomes Reported	Outcome Assessment
Creavin et al., 2012 [14]	UK (CaPS)	NR	Research Cohort	Community	*N* = 1,131	0.0%	57.7 (53.6–61.4)§§***	Up to 21***	NCEP-ATP III	All-cause dementia	Adjudicated based on DSM-IV, NINCDS-ADRDA, NINDS-AIREN
Exalto et al., 2015 [6]	Netherlands (ADC)	NR	Research Cohort	Memory clinic	*N* = 59	39.5%^#^	66.7 (9.7)^#^	3.4 (1.1)	NCEP-ATP III revised AHA/NHLBI	All-cause dementia	NR
Fan et al., 2017 [7]	Taiwan (TwSHHH)	100% Asian [30]	Research Cohort	Community	*N* = 3,458	53.0%	54.5 (10.7)^†^	9.2^||^	NCEP-ATP III revised AHA/NHLBI	All-cause dementia	Medical records, ICD-9-CM: 290.0, 290.1x, 290.2x, 290.3, 290.4x, 294.1, 331.0, 331.1, 331.2
Forti et al., 2010 [8]	Italy (CSBA)	NR	Research Cohort	Community	Age < 75 years: *N* = 466 Age ≥ 75 years: *N* = 283	Age < 75 years: 51.3% Age ≥ 75 years: 56.9%	Age < 75 years: 69.3 (3.0)^†^ Age ≥ 75 years: 79.8 (3.8)^†^	3.9 (0.8)	NCEP-ATP III	All-cause dementia AD VaD	Adjudicated based on DSM-IV, NINCDS-ADRDA, NINDS-AIREN [31]
Lee et al., 2020 [9]	South Korea (KNHI)	100% Asian [32]	Medical Records Cohort	Community	*N* = 4,106,590	45.5%	55.8 (10.1)	4.9 (NR)	Harmonized Criteria	All-cause dementia AD VaD	Neuropsychological exam, antidementia drug prescriptions, and medical records (ICD-10: AD [F00 or G30], VD [F01], or other dementia [F02, F03, G23.1 or G31])
Ng et al., 2016 [11]	Singapore (SLAS)	100% Asian	Research Cohort	Community	*N* = 1,936	64.8%^*^	64.9 (6.8)^*^	3.8 (NR) §§	IDF	All-cause dementia	Adjudicated based on DSM-IV
Muller et al., 2007 [10]	USA (Medicare – Manhattan)	29.9% White 30.8% African American 39.3% Hispanic	Medical Records Cohort	Community	*N* = 1,833	67.3%	76.1 (6.0)	4.4 (2.5)	NCEP-ATP III	All-cause dementia AD VaD	Adjudicated based on DSM-IV, CDR ≥1, NINCDS-ADRDA
Peng et al., 2018 [29]	China (Multicentre study)	100% Asian	Research Cohort	Community	*N* = 787	43.1%	67.1 (7.2)	5 (NR)	NCEP-ATP III	PDD	Adjudicated based on Movement Disorder Task Force (2007) criteria^##^
Raffaitin et al., 2009 [12]	France (3 C Study)	NR	Research Cohort	Community	*N* = 7,077	61%^††^	73.4 (4.9)^††^	Up to 4 (NR)	NCEP-ATP III	All-cause dementia AD VaD	Adjudicated based on DSM-IV, NINCDS-ADRDA, Hachinski score, MRI, history of vascular disease
Solfrizzi et al., 2010 [13]	Italy (ILSA)	NR	Research Cohort	Community	*N* = 2,097	47.3%	72.9 (5.6)	Up to 3.5 (NR)	NCEP-ATP III	All-cause dementia AD VaD Other dementias^^#^	Adjudicated based on DSM-III-R, NINCDS-ADRDA, and ICD-10
Machado-Fragua et al., 2022 [16]	UK (Whitehall II)	*Age < 60*: White: 90.1% Non-white: 9.9% *Age 60–69*: White:91.6% Non-white: 8.4% *Age ≥ 70*: White: 90.3% Non-white: 9.7%	Research Cohort	Community	*Age < 60*: *N* = 7,265 *Age 60–69*: *N* = 6,660 *Age ≥ 70*: *N* = 3,608	*Age < 60*: 30.5% *Age 60–69*: 29.3% *Age ≥ 70*: 29.4%	*Age < 60*: 55.1 (2.9) *Age 60–69*: 65.0 (1.5) *Age ≥ 70*: 73.9 (1.9)	*Age < 60*: 19.6 (5.9) *Age 60–69*: 10.9 (5.8) *Age ≥ 70*: 5.7 (3.2)	Harmonized Criteria	All-cause dementia	Medical records, ICD-10: F00-F03, F05.1, G30, G31
Qureshi et al., 2023 [27]	UK (UK Biobank)	White: 96.8% Non-white: 2.7% Missing: 0.5%	Research Cohort	Community	*N* = 176,249	52.2%	64.1 (2.9)	11.8 (2.2)	Harmonized Criteria	All-cause dementia	Medical records, ICD-9: 331.0, 290.4, 331.1, 290.2, 290.3, 291.2, 294.1, 331.2, 331.5; ICD-10: F00, F00.0, F00.1, F00.2, F00.9, G30, G30.0, G30.1, G30.8, G30.9, F01, F01.0, F01.1, F01.2, F01.3, F01.8, F01.9, I67.3, F02.0, G31.0, A81.0, F02, F02.1, F02.2, F02.3, F02.4, F02.8, F03, F05.1, F10.6, G31.1, G31.8
Qureshi et al., 2024 [28]	UK (EPIC-Norfolk)	White: 99.7% Non-white: 0.3%	Research Cohort	Community	*N* = 20,150	54.0%	62.6 (7.5)	18.8 (6.3)	Harmonized Criteria	All-cause dementia	Medical records, ICD-10: F00, G30, F00.0, G30.0, F00.1, G30.1, F00.2, G30.8, F00.9, G30.9, F01, F01.0, F01.1, F01.2, F01.8, F01.9, F02, F02.0, F02.1, F02.2, F02.3, F02.8, G31.0, G31.8, F03, F05.1, F10.7
Ekram et al., 2023 [15]	Australia & USA (ASPREE trial)	White: 91.2%^$#^ Non-white: 8.8%^$#^	Research Cohort	Community	*N* = 16,965	56.3%^$#^	75.1 (4.5)^$#^	4.7 (3.6–5.7)^§§ &^	ACC/AHA 2018 Guideline	All-cause dementia	Adjudicated based on DSM-IV

CaPS: Caerphilly Prospective Study, ADC: Amsterdam Dementia Cohort, TwSHHH: Taiwanese Survey on Prevalence of Hypertension Hyperglycemia and Hyperlipidemia, TNHIRD: Taiwan National Health Insurance Research Database, CSBA: Conselice Study of Brain Ageing, KNHI: Korean National Health Insurance Service - National Health Information Database, SLAS: Singapore Longitudinal Ageing Study, 3C Study: Three-City study, ILSA: Italian Longitudinal Study on Ageing, MetS: Metabolic syndrome, NCEP-ATP III: National Cholesterol Education Program - Adult Treatment Panel III, AHA/NHLBI: American Heart Association and the National Heart, Lung, and Blood Institute, IDF: International Diabetes Foundation, DSM: Diagnostic and Statistical Manual of Mental Disorders, NINCDS-ADRDA: National Institute of Neurological and Communicative Disorders and Stroke - Alzheimer’s Disease and Related Disorders Association, NINDS-AIREN: National Institute of Neurological Disorders and Stroke (NINDS) and the Association Internationale pour la Recherche et l’Enseignement en Neurosciences (AIREN), NR: Not reported, ICD-9: International Classification of Diseases, Ninth Revision, ICD-10: International Classification of Diseases, Tenth Revision CDR: Clinical Dementia Rating, SD: Standard deviation, AD: Alzheimer’s disease, VaD: Vascular dementia, PDD: Parkinson’s disease dementia; EPIC: European Prospective Investigation into Cancer, ASPREE: ASPirin in Reducing Events in the Elderly, ACC/AHA: American College of Cardiology/American Heart Association Task Force Guideline

§§: Median (Interquartile Range), #: Reported for 86 participants, *: Reported for 1,519 participants (primary sample), ***: Among phase 2 participants (n=2,398) - chosen based on balance between longest follow-up and largest sample size, †: Combined mean and SD,||: Calculated based on other available data. ††: Reported for 7,087 participants, &: Additional information provided by the authors, ^#: Defined as dementia in other diseases classified elsewhere (e.g., dementia in Pick’s disease, Creutzfeldt - Jakob disease, Huntington’s disease, Parkinson’s disease, human immunodeficiency virus [HIV], or other disease); $#: Among primary sample of 18,264 participants, &: Additional information provided by the authors

Mean baseline age varied across the studies; one study exclusively included participants aged < 65 years [14]eight studies included both participants aged < 65 and 65 + years [6, 7, 9, 11, 16, 27–29], and five studies exclusively included participants aged 65 + years.[8, 10, 12, 13, 15] The proportion of females included across studies ranged between 29% [16] to 67% [10], and one study included males only [14]. Follow-up length also varied considerably, ranging from a mean of 3.4 years [6] to up to 21 years [14]. Age and sex/gender were adjusted in the multivariate analysis for all but one study (which included males only) [14]. Eleven studies included adjustment for smoking status [7, 9–11, 13–16, 27–29], ten adjusted for education [8, 10–13, 15, 16, 27–29] nine for alcohol consumption [7, 9–11, 13–16, 27–29], five for *APOE*-ε4 carrier status [8, 10, 11, 27, 28] and three adjusted for socioeconomic status (defined as social class or based on the Townsend deprivation index score) [14, 27, 28]. 

### Risk of bias and quality assessment

Among the 14 studies, three were rated as ‘strong’ quality [8, 13, 28] nine as ‘moderate’ [6, 10–12, [14–16, 27, 29] and ‘two’ as weak [7, 9] (Table S4). Studies with a moderate rating were subject to selection bias (n = 7) [10–12, 15, 16, 27, 29], lack of sufficient confounder adjustment (n = 1) [6] or were subject to potential attrition (n = 1).[14] Studies with a weak rating did not describe the validity and reliability of data collection methods (n = 2) or exhibited a lack of sufficient confounder adjustment (n = 2).[7, 9]

### Meta-analysis eligibility

Four of 14 studies could not be included in meta-analyses as the exposure definition or reported effect estimates were not directly comparable with other studies [7, 14, 15, 29]. Therefore, a total of 10 studies were eligible for the meta-analyses [6, 8–13, 16, 27, 28]. Among these 10 studies, one study [8] reported two separate effect estimates corresponding to different and non-overlapping populations that were analysed separately (i.e., individuals aged < 75 and those aged ≥ 75) justifying the inclusion of both estimates in meta-analyses.

### MetS and all-cause dementia

**Fig. 2:**
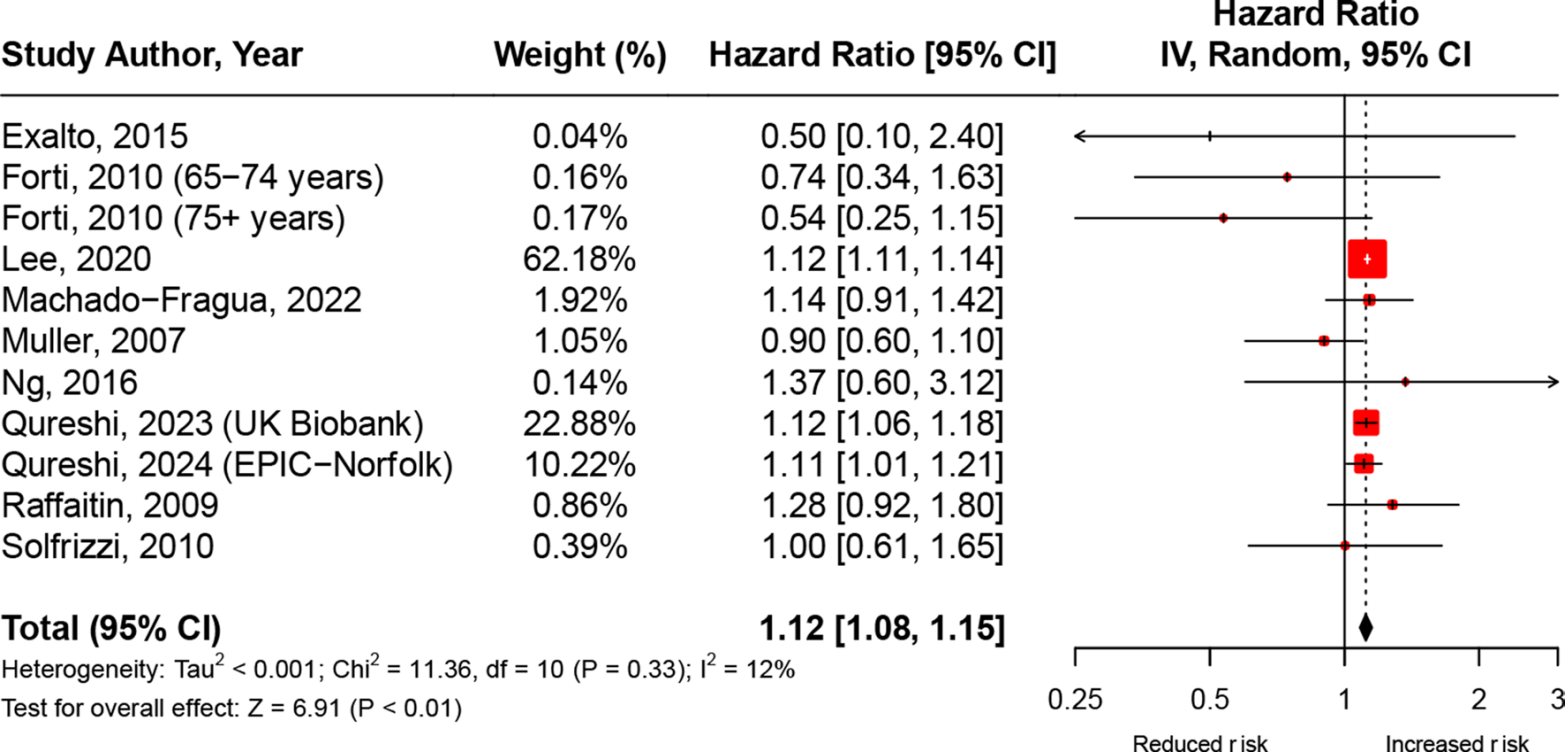
Meta-analysis of the hazard ratios of the association between MetS and incident all-cause dementia Abbreviations: CI = Confidence Interval; df = Degrees of Freedom. Note: Pooled estimates were calculated using random-effects meta-analysis models

All-cause dementia was reported in thirteen studies [6–16, 27, 28] (Table S5); eight reported a positive association [7, 9, 11, 12, 14, 16, 27, 28] among which four reached statistical significance [7, 9, 27, 28]. The meta-analysis of the association consisted of eleven effect estimates from ten studies [6, 8–13, 16, 27, 28]and indicated a higher risk of incident all-cause dementia in those with MetS compared to those without MetS (4,307,830 participants, 27,708 cases; pHR, 1.12, 95% CI: 1.08–1.15, *I*^*2*^ = 12.0%, Fig. 2).

The association between MetS and incident all-cause dementia remained consistent in subgroup analyses (Figure S1-S6) restricted to: (1) studies reporting results for those aged 65 + years (pHR, 1.08, 95% CI: 1.00-1.16, *I*^*2*^ = 15.0%); (2) studies that ascertained MetS using the 2009 Harmonized Criteria (pHR, 1.12, 95% CI: 1.11–1.14, *I*^*2*^ = 0.0%); (3) studies consisting of predominantly White populations (pHR, 1.11, 95% CI: 1.06–1.16, *I*^*2*^ = 0.0%); and (4) studies rated as ‘strong’ or ’moderate’ (i.e., excluding studies with a ‘weak’ global rating) based on risk of bias assessments (pHR, 1.09, 95% CI: 1.02–1.17, *I*^*2*^ = 17.0%). There was no statistically significant pooled association in subgroup analyses restricted to studies using the NCEP-ATP III criteria to ascertain MetS (pHR, 0.91, 95% CI: 0.70–1.18, *I*^*2*^ = 32.0%), and studies consisting of predominantly non-white populations (pHR, 1.03, 95% CI: 0.80–1.33, *I*^*2*^ = 57.0%).

### MetS and Alzheimer’s disease

**Fig. 3:**
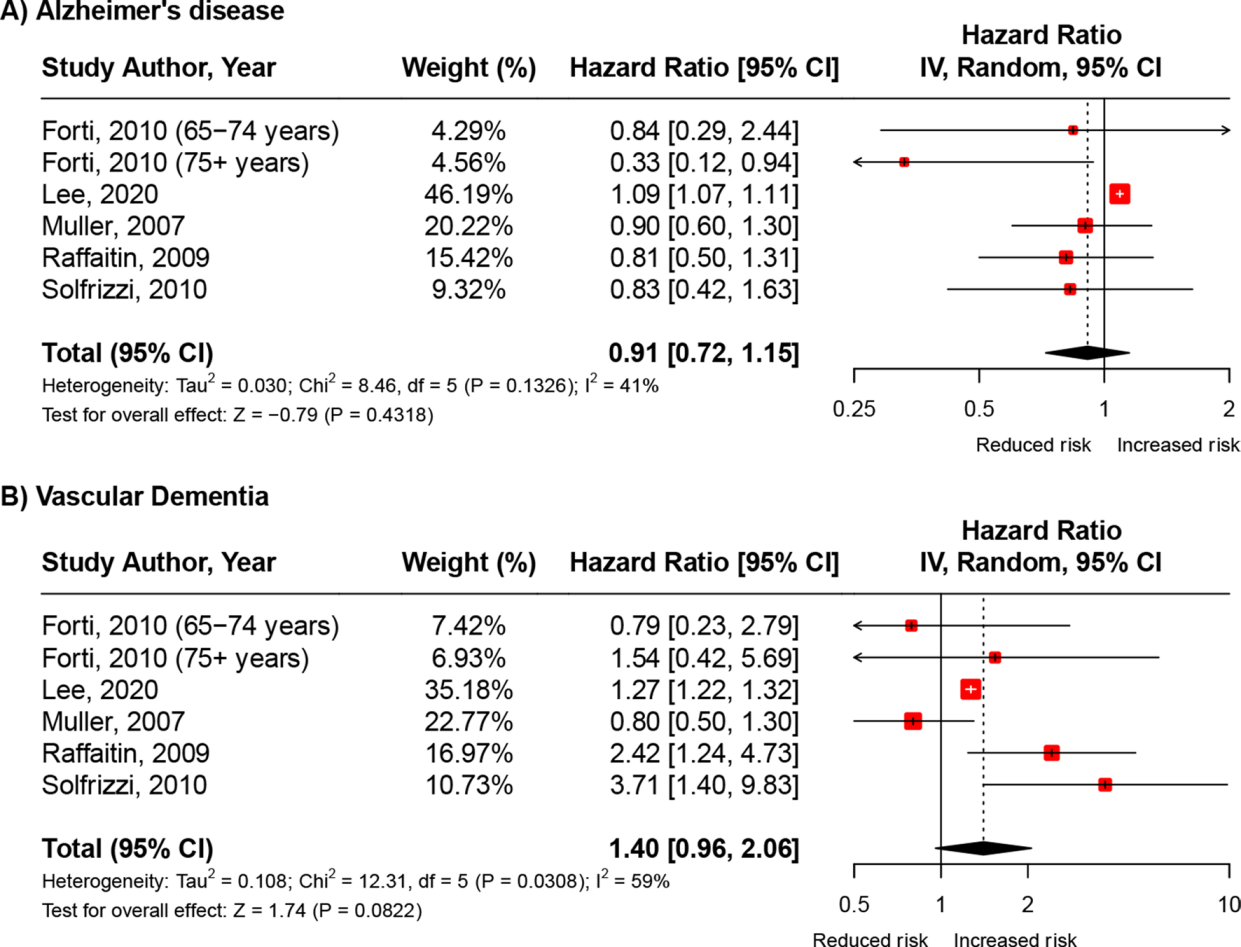
Meta-analysis of the hazard ratios of the association between **A**) MetS and incident Alzheimer’s disease and **B**) MetS and vascular dementia Abbreviations: CI = Confidence Interval; df = Degrees of Freedom. Note: Pooled estimates were calculated using random-effects meta-analysis models

Alzheimer’s disease was reported in five studies [8–10, 12, 13] (Table S6) among which results were mixed. One study reported a significant positive association [9]while another study reported a significant inverse association, specifically among participants aged 75 years or older [8]. The meta-analysis, which consisted of six effect estimates, found no overall association between MetS and risk of incident Alzheimer’s disease (4,109,436 participants, 14,890 cases; pHR, 0.91, 95% CI: 0.72–1.15, *I*^*2*^ = 41.0%, Fig. 3A).

These results remained similar in subgroup analyses (see Figures S1-S2, Figures S4-S6) restricted to: (1) studies reporting results for those aged 65 + years (pHR, 0.85, 95% CI: 0.65–1.11, *I*^*2*^ = 0.0%), (2) studies that ascertained MetS using the NCEP-ATP III criteria (pHR, 0.80, 95% CI: 0.62–1.04, *I*^*2*^ = 0.0%); (3) studies consisting of predominantly White populations (pHR, 0.74, 95% CI: 0.52–1.05, *I*^*2*^ = 0.0%); and (4) studies rated as ‘strong’ or ’moderate’ (i.e., excluding studies with a ‘weak’ global rating) based on risk of bias assessments (pHR, 0.80, 95% CI: 0.62–1.04, *I*^*2*^ = 0.0%). Similarly, subgroup analyses restricted to studies consisting of predominantly non-white populations showed comparable results, demonstrating no statistically significant association between MetS and incident Alzheimer’s disease (pHR, 1.08, 95% CI: 0.97–1.19, *I*^*2*^ = 0.0%). However, only two studies were included in this particular subgroup analysis [9, 10]. 

### MetS and vascular dementia

Vascular dementia was reported in five studies [8–10, 12, 13] (Table S7); four studies reported a positive association [8, 9, 12, 13] among which three reached statistical significance [9, 12, 13]. The meta-analysis, which consisted of six effect estimates indicated no overall significant association between MetS and risk of incident vascular dementia (4,109,436 participants, 2,642 cases; pHR, 1.40, 95% CI: 0.96–2.06, *I*^*2*^ = 59.0%, Fig. 3B).

These results remained similar in subgroup analyses (see Figures S1-S2, Figures S4-S6) restricted to: (1) studies reporting results for those aged 65 + years (pHR, 1.55, 95% CI: 0.71–3.39, *I*^*2*^ = 75.0%); (2) studies that ascertained MetS using the NCEP-ATP III criteria (pHR, 1.54, 95% CI: 0.79-3.00, *I*^*2*^ = 67.0%); and (3) studies rated as ‘strong’ or ’moderate’ (i.e., excluding studies with a ‘weak’ global rating) based on risk of bias assessments (pHR, 1.54, 95% CI: 0.79-3.00, *I*^*2*^ = 67.0%). Similarly, subgroup analyses restricted to studies consisting of predominantly non-white populations (pHR, 1.08, 95% CI: 0.71–1.65, *I*^*2*^ = 71.0%, P_Heterogeneity_ = 0.06) or predominantly White populations (pHR, 2.08, 95% CI: 1.18–3.67, *I*^*2*^ = 25.0%, P_Heterogeneity_ = 0.26) showed comparable results. Notably, greater heterogeneity was observed across studies consisting of predominantly non-white populations as compared to those of predominantly White populations.

### Other dementias

One study reported on Parkinson’s disease-dementia (Table S8) and found a significant positive association (Odds Ratio, 2.12, 95% CI: 1.57–2.83) [29]. 

### Sensitivity and exploratory meta-analyses

Additional sensitivity and exploratory meta-analyses were conducted to assess the robustness of findings and explore further potential sources of heterogeneity. When compared to the main meta-analyses (Figs. 2 and 3), findings from these additional analyses revealed that: (1) in meta-analyses excluding studies without clinical adjudication of dementia diagnosis, a weaker and non-significant association was observed for all-cause dementia, while similar patterns were observed for Alzheimer’s disease and vascular dementia (Figure S7); (2) in meta-analyses excluding studies adjusting for vascular intermediate outcomes (e.g., stroke or cardiovascular disease) in the relationship between MetS and vascular dementia, the pooled estimate remained consistent with the main findings (Figure S8); and (3) when restricting meta-analyses to studies conducted outside of the United States, pooled estimates remained similar across all outcomes of interest, although a slightly stronger and statistically significant association was observed for vascular dementia (Figure S9).

In further meta-analyses restricting to studies examining MetS trajectories over time, a significant positive association was observed between prolonged/persistent MetS and incident all-cause dementia (Figure S10). Finally, in meta-analyses of studies examining the relationship between the number of MetS components present and dementia risk, a dose-response relationship was observed, whereby an increasing number of MetS components was associated with a greater risk of incident all-cause dementia (Figure S11).

### Meta-regression analyses

The meta-regression analyses (Table 2) showed little evidence of effect modification based on the year of publication (adjusted *p* = 0.249), mean/median follow-up duration (adjusted *p* = 0.646), proportion of female participants among studies (adjusted *p* = 0.116), adjustment for *APOE*-ε4 carrier status (adjusted *p* = 0.225), ethnicity (predominantly White vs. predominantly non-white; adjusted *p* = 0.570), and geographic region (Western vs. non-Western; adjusted *p* = 0.345).^6,8–13,16,27,28^ However, there was some evidence for effect modification based on mean/median baseline age of participants included in the identified studies (adjusted *p* = 0.043), and studies using the NCEP-ATP III criteria to ascertain MetS (adjusted *p* = 0.019); specifically, studies with a higher baseline age tended to show a weaker association (Est: -0.003) between MetS and dementia risk, and studies using the NCEP-ATP III criteria (Est: -0.145) showed lower pooled effect estimates compared with other definitions. As only one study received a ‘weak’ quality assessment rating [9]meta-regression was not performed for the characteristic of “study quality/risk of bias assessment”, however, a sensitivity analysis excluding this weak study revealed similar findings to the main meta-regression results.

**Table 2:** Univariable meta-regression models to assess the influence of various study-level factors among articles investigating the association between MetS and risk of incident all-cause dementia (eligible results *N* = 11)

Study characteristic	Estimate	95% CI	Q (m)	*p* value	Adjusted p*
Publication year	0.004	-0.007–0.014	0.425	0.514	0.249
Mean/median follow-up (in years)	-0.001	-0.006–0.004	0.117	0.732	0.646
Mean/median age at baseline (in years)	-0.003	-0.008–0.002	1.036	0.309	0.043
Used NCEP-ATP III MetS criteria (yes vs. no)	-0.145	-0.336–0.046	2.212	0.137	0.019
% female sex	-0.002	-0.007–0.003	0.644	0.422	0.116
Adjusted for *APOE*-ε4 carrier status (yes vs. no)	-0.017	-0.064–0.030	0.500	0.480	0.225
Ethnicity (predominantly White vs. not)	-0.011	-0.057–0.036	0.197	0.657	0.570
Geographic region (Western vs. non-Western)	-0.015	-0.061–0.031	0.432	0.511	0.345

Abbreviations: NCEP-ATP III = National Cholesterol Education Program – Adult Treatment Panel III; Q (m) = Q for moderators; MetS = Metabolic Syndrome; APOE = Apolipoprotein-E

Note: Pooled estimates were calculated using random-effects meta-analysis models

*Using permutation test

### Publication bias

Based on funnel plots, there was a low likelihood of publication bias among studies investigating MetS in relation to all-cause dementia and vascular dementia (Figure S12). In contrast, there was some evidence for publication bias for Alzheimer’s disease. The Egger’s tests found a low likelihood of publication bias for all-cause dementia (*p* = 0.078, Table S9). Due to the limited number of studies (i.e., *n* < 10), Egger’s test was not calculated for Alzheimer’s disease or vascular dementia.

## Discussion

This updated systematic review and meta-analysis demonstrated MetS was associated with a significantly increased risk of developing incident all-cause dementia and which appeared to be restricted to vascular dementia. However, the association with vascular dementia was not statistically significant, likely reflecting the small sample size. In contrast to all-cause dementia, the majority of studies assessing dementia subtypes were published more than a decade ago with small case numbers, emphasising that further investigation of the associations between MetS and dementia subtypes is clearly warranted.

In the current meta-analysis, the magnitude of association between MetS and all-cause dementia risk was similar to a 2019 systematic review, albeit the previous study reported a non-statistically significant association (pHR, 1.12, 95% CI: 0.94–1.33, *I*^*2*^ = 28.0%).[3] In contrast, we observed a statistically significant association between MetS and all-cause dementia, which was likely due to the incorporation of new longitudinal studies increasing the number of cases and resulting in more precise effect estimates. In regards to Alzheimer’s disease, our findings are consistent with a 2021 meta-analysis which found no significant association among four longitudinal studies for incident Alzheimer’s disease [4] In regards to vascular dementia, the 2019 review found that MetS was associated with an increased risk of incident vascular dementia (pHR, 1.37, 95% CI: 1.01–1.86, *I*^*2*^ = 62.0%) based on pooled results from 4 studies [3] whereas in the current review, we observed a similar (but non-significant) magnitude of association between MetS and vascular dementia based on pooled results from 5 studies.

The relationship of MetS and all-cause dementia remained consistent in several subgroup analyses restricting to participants aged 65 + years, using different MetS criteria, using clinical adjudication for dementia ascertainment, excluding studies which adjusted for intermediate outcomes, restricting to studies with predominantly White populations, and after excluding studies with weak quality assessment ratings. No significant association was observed for any of the dementia outcomes when restricting to studies consisting of predominantly non-white populations. However, these findings should be interpreted with caution as only three studies for all-cause dementia [9–11]and two for Alzheimer’s disease [9, 10] and vascular dementia [9, 10] respectively, were conducted in non-white populations. Significant associations were observed when examining studies that used the 2009 Harmonized Criteria to define MetS, but not for those that used the NCEP-ATP III criteria; this finding suggests that some of the variation evident across studies may be influenced by differences in the MetS criteria adopted across studies. This could be due to differences in cut-offs for high blood glucose, or inclusion vs. exclusion of ethnic-specific cut-offs for waist circumference. We also found that (1) long-term exposure to MetS (based on studies which performed trajectory analyses) and (2) an increasing number of MetS components, was associated with an increased risk of all-cause dementia. These findings suggest that duration and intensity of exposure to MetS may affect risk of dementia. The associations were null when restricting to studies with adjudicated dementia diagnoses and without adjustment for vascular diseases, but these analyses consisted of a limited number of studies. These subgroup findings are important in highlighting gaps in the current evidence base, in particular the need to focus on diverse populations and clarity on how MetS is conceptualised in the context of dementia risk.

Findings from this study suggest that there may be distinct associations between MetS and different dementia subtypes. Although neither association reached statistical significance, the pooled HR for incident Alzheimer’s disease was 0.91 (95% CI: 0.72–1.15), suggesting a null association, while the pooled HR for vascular dementia was 1.40 (95% CI: 0.96–2.06), suggesting a potential positive association. These differences may be due to chance, or they may reflect the distinct pathophysiological mechanisms at play in the association between MetS and the risk of developing these different dementia subtypes. Alzheimer’s disease is known to be characterised by neurodegenerative changes within the brain, including amyloid depositions and neurofibrillary tangles, which are associated with cellular dysfunction and neuronal death [33, 34]. While there is some evidence to suggest that the mechanisms involved in MetS – such as insulin resistance, hyperinsulinemia, or inflammation – may contribute to Alzheimer’s disease pathology, there is stronger mechanistic evidence linking MetS to the development of vascular dementia, particularly due to the number of vascular-related components used to define a diagnosis of MetS [33, 34]. More specifically, the pathogenesis of vascular dementia is primarily driven by vascular risk factors (such as those inherent in MetS) which lead to cerebrovascular disease and reduced blood flow to the brain that, in turn, result in neurological injury and disruption of cognitive networks, eventually leading to the development of vascular dementia [35, 36]. 

A moderating association for age was observed in a meta-regression analysis, indicating that as age increases, the strength of the association between MetS and all-cause dementia slightly decreases. This is consistent with the 2024 Lancet Commission on dementia prevention, which proposed that the individual metabolic and vascular components of MetS are risk factors for dementia when exposed at mid- rather than late-life [1]. We also found that the use of the NCEP-ATP III criteria was associated with a slightly weaker reported association between MetS and all-cause dementia, implying that the choice of MetS criteria is important. Taken together, findings from these meta-regression models underscore the importance of considering age-specific differences as well as the importance of standardising methods to ascertain MetS when designing studies and interpreting data in context of the relationship between MetS and dementia risk.

This study is, to date, the largest and most comprehensive systematic review and meta-analysis of longitudinal studies investigating the relationship between MetS, incident all-cause dementia and dementia subtypes. However, this work also has several limitations. First, only studies published in English were included, which could limit the generalisability of the findings. Second, while a broad set of MetS criteria and dementia outcomes were captured, the minor inconsistences in both exposure and outcome definitions adopted within studies may still contribute to the heterogeneity observed in the findings limiting the overall comparability between included studies. Third, despite inclusion of recent data, the overall number of studies was still relatively small, which limit the interpretation and generalisability of the findings, particularly for dementia subtypes. Fourth, meta-regression analyses and Egger’s test could not be conducted for the dementia subtypes, again due to the limited the number of studies examining these outcomes. Fifth, the reported estimates across studies may not reflect the true magnitude of the association between MetS and dementia due to the potential for reverse causation, selection bias, or potential exposure/outcome misclassification, although this is a limitation of the original studies rather than the review [37]. 

## Conclusions

In conclusion, this systematic review and meta-analysis found evidence that MetS is associated with the risk of developing all-cause dementia and that this association may differ by disease subtype, being stronger for vascular dementia than Alzheimer’s disease. These differences align with the possible mechanisms that might explain the pathogenesis of these subtypes. However, further large-scale studies with long follow-up and in diverse populations are required to fully understand these relationships and better characterise the associations with different dementia subtypes.

## Electronic supplementary material

Below is the link to the electronic supplementary material.

Supplementary Material 1

## Data Availability

No datasets were generated or analysed during the current study.

## Abbreviations

APOE: Apolipoprotein-E
CI: Confidence Interval
HR: Hazard Ratio
MetS: Metabolic Syndrome
NCEP-ATP III: National Cholesterol Education Program - Adult Treatment Panel III
pHR: Pooled Hazard Ratio
