# Supplementary material for: Metabolic syndrome and risk of incident all-cause dementia, Alzheimer’s disease and vascular dementia: a systematic review and meta-analysis of longitudinal studies
Source: Alzheimers Res Ther. 2025 Aug 23;17:198. doi: 10.1186/s13195-025-01825-4 (PMC12374402; doi:10.1186/s13195-025-01825-4)
Supplement: Supplementary file 1 — Supplementary Material 1 [file 13195_2025_1825_MOESM1_ESM.docx]

### Methods S1: Systematic Review Protocol

**Review question:** Do prospective studies suggest an association between metabolic syndrome (MetS) and risk of incident all-cause dementia or key dementia subtypes?

**Population:** Adults (≥18 years)

**Exposure:** Prevalent MetS

**Comparators:** No prevalent MetS

**Outcomes:** Incident all-cause dementia or key dementia subtypes (e.g., Alzheimer's disease, vascular dementia, Lewy body Dementia, and frontotemporal dementia)

**Search strategy:**

- Selection of relevant MetS (exposure) search terms will be informed by:
  - Key papers discussing standardised definitions of MetS^1-8^;
  - MetS review articles^9-21^;
  - Search strategies of the top 50 most-cited MetS systematic reviews (identified via Web of Science)^22-71^
- Dementia (outcome) search terms will be informed by:
  - Review articles discussing dementia and key dementia subtypes^72-75^;
  - Search strategies used in previous systematic reviews where all-cause dementia and/or key dementia subtypes are the main outcome^76-126^
- Study design search terms will be informed by:
  - Search strategy used in a previous systematic review of prospective studies^126^;
  - The Scottish Intercollegiate Guidelines Network (SIGN) search filters for systematic reviews^127^

Our search strategy will include:

- Searches will be developed for MEDLINE, Embase, and PsycINFO and will incorporate relevant subject headings and free-text terms informed by the approach outlined above. All searches will be peer-reviewed by an experienced librarian
- Backward and forward citation searches of included studies via Web of Science
- Automated search alerts set up in OvidSP (for Medline, Embase and PsycINFO databases) to receive regular email updates when new studies are published after the search date

**Study selection criteria:**

**Inclusion criteria:**

- Participants must be 18 years or older at study baseline
- Prospective studies examining the association between metabolic syndrome and incident all-cause dementia or key dementia subtypes
- MetS defined as follows: (1) self-reported diagnosis, or diagnosis confirmed through medical history (i.e. medical records); or (2) any combination of ≥ 3 out of the following 6 components (or an appropriate proxy indicator for any component): obesity, elevated fasting blood glucose, elevated triglyceride levels, reduced high-density lipoprotein cholesterol, elevated blood pressure, or microalbuminuria*
- Studies must report outcomes as incident dementia or key dementia subtypes (e.g., Alzheimer’s disease, vascular dementia, Lewy body dementia, and frontotemporal dementia)
- Publications must be written in English

**Exclusion criteria:**

- Studies which do not define the presence or absence of MetS using the following 5 components (or an appropriate proxy indicator): obesity, elevated fasting blood glucose, elevated triglyceride levels, reduced high-density lipoprotein cholesterol, elevated blood pressure
- Studies which define the presence or absence of MetS using a combination of measures outside of the following 6 components: obesity, elevated fasting blood glucose, elevated triglyceride levels, reduced high-density lipoprotein cholesterol, elevated blood pressure, and microalbuminuria*
- Studies with no comparison group or comparison group other than no metabolic syndrome
- Studies with outcomes that are not directly dementia-related (e.g. neuroimaging or biomarkers)
- Studies using only a single cognitive instrument or self-reported dementia to define incident dementia or key dementia subtypes
- Animal studies
- Case reports, narrative reviews, letters, editorials, opinions, book chapters
- Conference abstracts
- Duplicate publications using the same data

*Note: microalbuminuria was included in the list of components to capture studies that used the original World Health Organisation standardised criteria for defining MetS, alongside other established MetS criteria.

**Study selection:** Two reviewers (DQ & EK) will independently screen titles and abstracts
based on the inclusion/exclusion criteria. The same two reviewers will also independently review the full-texts of potentially relevant studies. Any discrepancies will be resolved by discussion, if necessary, with a third reviewer (TJL).

**Risk of bias (quality) assessment:** Two reviewers (DQ & TJL) will independently assess the risk of bias using the Effective Public Health Practice Project (EPHPP) Quality Assessment Tool for Quantitative Studies.^128^ The components of the tool will be rated as outlined in previously described methods (described further below).^126^ Any discrepancies will be resolved by discussion, if necessary, with a third reviewer (EK).

The Quality Assessment components will be rated individually as strong, moderate or weak as follows:

1. **Selection bias:** will be rated as strong if study participants are likely to be representative of the target population and the participation rate at baseline is greater than 80%, as moderate if participants are at least somewhat likely to be representative of the target population and the participation rate at baseline is 60-79%, and as weak if participants are not likely to be representative of the target population or the participation rate at baseline is less than 60% or selection and participation rate at baseline are not described.
2. **Design:** will be rated as strong for randomised controlled trials and controlled clinical trials, as moderate for cohort studies, case control studies or interrupted time series and as weak for any other method or if method is not described.
3. **Confounders:** We consider adjusting analyses for sociodemographic confounders (age, sex, education and / or socioeconomic status, and ethnicity in case of a heterogenous sample) as essential. Studies will be rated as strong if analyses are adjusted for sociodemographic and at least three additional confounders and as moderate if adjustment strategy includes sociodemographic and one or two additional confounders. Studies will receive a weak rating if only sociodemographic or not all relevant sociodemographic confounders are adjusted for.
4. **Blinding:** will be rated as strong if the outcome assessor is not aware of participants’ MetS status and participants are not aware of the research question / assessment of MetS for research purposes, as moderate if the outcome assessor is not aware of participants’ MetS status or participants are not aware of the research question / assessment of MetS for research purposes or blinding is not described and as weak if the outcome assessor is aware of participants’ MetS status and participants are aware of the research question / assessment of MetS for research purposes.
5. **Data Collection Methods:** will be rated as strong if data collection tools have been shown or are widely known to be valid and reliable, as moderate if data collection tools have been shown or are widely known to be valid but not reliable or reliability is not described and as weak if data collection tools have been shown or are widely known not to be valid or both validity and reliability are not described.
6. **Withdrawals and Drop-outs (Attrition Bias):** will be rated as strong if the follow-up rate is at least 80%, as moderate if the follow-up rate is 60-79% or follow-up rate cannot be assessed due to study design (not applicable) and as weak if the follow-up rate is less than 60% or withdrawals and drop-outs are not described.

**Data extraction:**

One reviewer (DQ) will extract key data from each included study (e.g. study author, publication year, country/region, data source, race and/or ethnicity, study design, setting, analytic sample size, proportion of females, year(s) in which baseline data were collected, baseline age, follow-up length, MetS criteria, dementia criteria, number and percentage of MetS cases, number and percentage of incident dementia cases, reported adjusted effect estimates for dementia and/or key subtypes, and adjustment variables). Data extraction will be checked by a second reviewer (EK or TJL). Any discrepancies will be resolved by discussion, if necessary with a third reviewer (EK or TJL).

Corresponding authors of included studies will be contacted for clarification (if necessary) or to obtain additional data that has not been fully reported.

**Evidence synthesis methods:**

The identified evidence on the associations between MetS and all-cause dementia and key dementia subtypes will be synthesized narratively and using random effects meta-analysis if appropriate. Heterogeneity between studies will be investigated using the x^2^ test for homogeneity and I^2^ statistic, and meta-regression if appropriate.

Small study effects will also be explored, including publication bias using funnel plots and Egger’s statistic if appropriate; specifically, funnel plots will be used to investigate publication bias if there are at least ten studies included in a meta-analysis.^129^

Secondary and sensitivity analyses may be performed to assess potential differences by exposure definitions, follow-up length, dementia subtypes, study quality, and other important measures.

### Table S1: MEDLINE Search Strategy

| **#** | **Query** |
| --- | --- |
| 1 | exp Metabolic Syndrome/ |
| 2 | exp Insulin Resistance/ |
| 3 | ((metabolic* or cardiometabolic* or dysmetabolic* or plurimetabolic* or atherometabolic*) adj3 (syndrome* or disorder* or risk factor* or abnormalit* or dysfunction*)).tw. |
| 4 | MetS*.tw. |
| 5 | (insulin* adj3 resistan*).tw. |
| 6 | ((reaven* or atherothrombogenic or proatherogenic or obesity dyslipid?emi* or central fat or central obesity or central adiposity or visceral fat or visceral obesity or visceral adiposity or new world or hyperinsulin* or glucose intolerance or "glucose intolerance hypertension obesity" or GHO or "hypertension-hyperglyc?emia-hyperuric?emia" or "dyslipid?emia insulin resistance obesity and high blood pressure" or DROP) adj syndrome*).tw. |
| 7 | syndrome x.tw. |
| 8 | android obesity.tw. |
| 9 | hypertriglycerid?emi* waist.tw. |
| 10 | "syndrome of affluence".tw. |
| 11 | ((death or deadly) adj quartet).tw. |
| 12 | "upper-body obesity glucose intolerance hypertriglycerid?emia and hypertension".tw. |
| 13 | ("coronary artery disease hypertension adult onset diabetes obesity and stroke" or "CHAOS").tw. |
| 14 | metabolic trisyndrome.tw. |
| 15 | 1 or 2 or 3 or 4 or 5 or 6 or 7 or 8 or 9 or 10 or 11 or 12 or 13 or 14 |
| 16 | exp Dementia/ |
| 17 | dement*.tw. |
| 18 | alzheimer*.tw. |
| 19 | 16 or 17 or 18 |
| 20 | exp Follow-Up Studies/ |
| 21 | exp Prospective Studies/ |
| 22 | exp Risk Factors/ |
| 23 | exp Longitudinal Studies/ |
| 24 | exp Proportional Hazards Models/ |
| 25 | exp Incidence/ |
| 26 | follow up.tw. |
| 27 | prospective*.tw. |
| 28 | longitudinal*.tw. |
| 29 | predict*.tw. |
| 30 | inciden*.tw. |
| 31 | determinant*1.tw. |
| 32 | hazard*1.tw. |
| 33 | risk.tw. |
| 34 | 20 or 21 or 22 or 23 or 24 or 25 or 26 or 27 or 28 or 29 or 30 or 31 or 32 or 33 |
| 35 | 15 and 19 and 34 |

### Table S2: Embase Search Strategy

| **#** | **Query** |
| --- | --- |
| 1 | exp metabolic syndrome X/ |
| 2 | exp cardiometabolic risk/ |
| 3 | exp insulin resistance/ |
| 4 | ((metabolic* or cardiometabolic* or dysmetabolic* or plurimetabolic* or atherometabolic*) adj3 (syndrome* or disorder* or risk factor* or abnormalit* or dysfunction*)).tw. |
| 5 | MetS*.tw. |
| 6 | (insulin* adj3 resistan*).tw. |
| 7 | ((reaven* or atherothrombogenic or proatherogenic or obesity dyslipid?emi* or central fat or central obesity or central adiposity or visceral fat or visceral obesity or visceral adiposity or new world or hyperinsulin* or glucose intolerance or "glucose intolerance hypertension obesity" or GHO or "hypertension-hyperglyc?emia-hyperuric?emia" or "dyslipid?emia insulin resistance obesity and high blood pressure" or DROP) adj syndrome*).tw. |
| 8 | syndrome x.tw. |
| 9 | android obesity.tw. |
| 10 | hypertriglycerid?emi* waist.tw. |
| 11 | "syndrome of affluence".tw. |
| 12 | ((death or deadly) adj quartet).tw. |
| 13 | "upper-body obesity glucose intolerance hypertriglycerid?emia and hypertension".tw. |
| 14 | ("coronary artery disease hypertension adult onset diabetes obesity and stroke" or "CHAOS").tw. |
| 15 | metabolic trisyndrome.tw. |
| 16 | 1 or 2 or 3 or 4 or 5 or 6 or 7 or 8 or 9 or 10 or 11 or 12 or 13 or 14 or 15 |
| 17 | exp dementia/ |
| 18 | dement*.tw. |
| 19 | alzheimer*.tw. |
| 20 | 17 or 18 or 19 |
| 21 | exp cohort analysis/ |
| 22 | exp major clinical study/ |
| 23 | exp follow up/ |
| 24 | exp prospective study/ |
| 25 | exp risk factor/ |
| 26 | exp longitudinal study/ |
| 27 | exp proportional hazards model/ |
| 28 | exp incidence/ |
| 29 | follow up.tw. |
| 30 | prospective*.tw. |
| 31 | longitudinal*.tw. |
| 32 | predict*.tw. |
| 33 | inciden*.tw. |
| 34 | determinant*1.tw. |
| 35 | hazard*1.tw. |
| 36 | risk.tw. |
| 37 | 21 or 22 or 23 or 24 or 25 or 26 or 27 or 28 or 29 or 30 or 31 or 32 or 33 or 34 or 35 or 36 |
| 38 | 16 and 20 and 37 |

### Table S3: PsycINFO Search Strategy

| **#** | **Query** |
| --- | --- |
| 1 | exp Metabolic Syndrome/ |
| 2 | ((metabolic* or cardiometabolic* or dysmetabolic* or plurimetabolic* or atherometabolic*) adj3 (syndrome* or disorder* or risk factor* or abnormalit* or dysfunction*)).tw. |
| 3 | MetS*.tw. |
| 4 | (insulin* adj3 resistan*).tw. |
| 5 | ((reaven* or atherothrombogenic or proatherogenic or obesity dyslipid?emi* or central fat or central obesity or central adiposity or visceral fat or visceral obesity or visceral adiposity or new world or hyperinsulin* or glucose intolerance or "glucose intolerance hypertension obesity" or GHO or "hypertension-hyperglyc?emia-hyperuric?emia" or "dyslipid?emia insulin resistance obesity and high blood pressure" or DROP) adj syndrome*).tw. |
| 6 | syndrome x.tw. |
| 7 | android obesity.tw. |
| 8 | hypertriglycerid?emi* waist.tw. |
| 9 | "syndrome of affluence".tw. |
| 10 | ((death or deadly) adj quartet).tw. |
| 11 | "upper-body obesity glucose intolerance hypertriglycerid?emia and hypertension".tw. |
| 12 | ("coronary artery disease hypertension adult onset diabetes obesity and stroke" or "CHAOS").tw. |
| 13 | metabolic trisyndrome.tw. |
| 14 | 1 or 2 or 3 or 4 or 5 or 6 or 7 or 8 or 9 or 10 or 11 or 12 or 13 |
| 15 | exp Dementia/ |
| 16 | dement*.tw. |
| 17 | alzheimer*.tw. |
| 18 | 15 or 16 or 17 |
| 19 | followup studies/ |
| 20 | prospective studies/ |
| 21 | risk factors/ |
| 22 | exp longitudinal studies/ |
| 23 | follow up.tw. |
| 24 | prospective*.tw. |
| 25 | longitudinal*.tw. |
| 26 | predict*.tw. |
| 27 | inciden*.tw. |
| 28 | determinant*1.tw. |
| 29 | hazard*1.tw. |
| 30 | risk.tw. |
| 31 | 19 or 20 or 21 or 22 or 23 or 24 or 25 or 26 or 27 or 28 or 29 or 30 |
| 32 | 14 and 18 and 31 |

| Table S4: Quality assessment (risk of bias) ratings for all studies included in the systematic review   \| **Study Author, Publication Year** \| **Selection Bias** \| **Study Design** \| **Confounders** \| **Blinding** \| **Data Collection Methods** \| **Withdrawals and Drop-outs** \| **Global Rating** \| \| --- \| --- \| --- \| --- \| --- \| --- \| --- \| --- \| \| **Creavin et al., 2012^14^** \| STRONG \| MODERATE \| STRONG \| MODERATE \| STRONG \| WEAK \| MODERATE \| \| **Ekram et al., 2023^15^** \| WEAK \| MODERATE \| STRONG \| STRONG \| STRONG \| STRONG \| MODERATE \| \| **Exalto et al., 2015^6^** \| MODERATE \| MODERATE \| WEAK \| MODERATE \| STRONG \| STRONG \| MODERATE \| \| **Fan et al., 2017^7^** \| MODERATE \| MODERATE \| WEAK \| MODERATE \| WEAK \| STRONG \| WEAK \| \| **Forti et al., 2010^8^** \| MODERATE \| MODERATE \| STRONG \| MODERATE \| STRONG \| MODERATE \| STRONG \| \| **Lee et al., 2020^9^** \| MODERATE \| MODERATE \| WEAK \| MODERATE \| WEAK \| MODERATE \| WEAK \| \| **Machado-Fragua et al., 2022^16^** \| WEAK \| MODERATE \| STRONG \| MODERATE \| STRONG \| STRONG \| MODERATE \| \| **Muller et al., 2007^10^** \| WEAK \| MODERATE \| STRONG \| MODERATE \| STRONG \| STRONG \| MODERATE \| \| **Ng et al., 2016^11^** \| WEAK \| MODERATE \| STRONG \| MODERATE \| STRONG \| STRONG \| MODERATE \| \| **Peng et al., 2018^29^** \| WEAK \| MODERATE \| STRONG \| MODERATE \| STRONG \| STRONG \| MODERATE \| \| **Qureshi et al., 2023 (UK Biobank)^27^** \| WEAK \| MODERATE \| STRONG \| MODERATE \| STRONG \| STRONG \| MODERATE \| \| **Qureshi et al., 2024 (EPIC-Norfolk)^28^** \| STRONG \| MODERATE \| STRONG \| MODERATE \| STRONG \| STRONG \| STRONG \| \| **Raffaitin et al., 2009^12^** \| WEAK \| MODERATE \| MODERATE \| MODERATE \| STRONG \| STRONG \| MODERATE \| \| **Solfrizzi et al., 2010^13^** \| STRONG \| MODERATE \| STRONG \| MODERATE \| STRONG \| MODERATE \| STRONG \| |
| --- | --- | --- | --- | --- | --- | --- | --- | --- | --- | --- | --- | --- | --- | --- | --- | --- | --- | --- | --- | --- | --- | --- | --- | --- | --- | --- | --- | --- | --- | --- | --- | --- | --- | --- | --- | --- | --- | --- | --- | --- | --- | --- | --- | --- | --- | --- | --- | --- | --- | --- | --- | --- | --- | --- | --- | --- | --- | --- | --- | --- | --- | --- | --- | --- | --- | --- | --- | --- | --- | --- | --- | --- | --- | --- | --- | --- | --- | --- | --- | --- | --- | --- | --- | --- | --- | --- | --- | --- | --- | --- | --- | --- | --- | --- | --- | --- | --- | --- | --- | --- | --- | --- | --- | --- | --- | --- | --- | --- | --- | --- | --- | --- | --- | --- | --- | --- | --- | --- | --- | --- |

### Table S5: Results of included studies for the association between MetS and incident all-cause dementia

| **Study author, publication year** | **Number and % of MetS cases** | **Number and % of incident all-cause dementia** | **Effect estimate (95% CI)** | **Confounder adjustments** |
| --- | --- | --- | --- | --- |
| **Creavin et al., 2012^130^** | NR | NR | OR: 1.54 (0.89 - 2.66)^#^  p = NR | Age, social class, smoking, alcohol intake, National Adult Reading Test |
| **Exalto et al., 2015^131^** | 23 (39.0%) | 10 (16.9%) | HR: 0.50 (0.10 - 2.40)  p = NR | Age, gender |
| **Fan et al., 2017^132^** | 1,122 (32.4%) | 76 (2.2%) | Persistent MetS, HR: 1.12 (0.57 - 2.21), p = 0.752  Improved MetS, HR: 0.88 (0.27 - 2.88), p = 0.829  Worsened MetS, HR: 2.22 (1.32 - 3.72), p = 0.003 | Age, sex, smoking, alcohol intake, physical activity, betel quid chewing, heart disease, stroke, depression |
| **Forti et al., 2010^133^** | <75 years: 133 (28.5%)  ≥75 years: 63 (22.3%) | <75 years: 35 (7.5%)  ≥75 years: 52 (18.3%) | <75 years: HR: 0.74 (0.34 - 1.63)  p = NR  ≥75 years: HR: 0.54 (0.25 - 1.15)  p = 0.11 | Age, sex, education, APOE-ε4 carrier status, physical activity, hyperhomocysteinemia, and inflammation status, history of cardiovascular disease, history of stroke |
| **Lee et al., 2020^134^** | 1,880,175 (45.8%) | ~19,286 (0.5%)^\|\|^ | HR: 1.12 (1.11 - 1.14)  p = NR | Age, sex, smoking, alcohol intake, physical activity, stroke, depression, chronic kidney disease |
| **Ng et al., 2016^135^** | 443(29.2%)^*^ | 26 (1.3%) | HR: 1.37 (0.60 - 3.12), p = 0.49 | Sex, age, education, APOE-ε4 carrier status, smoking, and physical/social/productive activities score |
| **Muller et al., 2007^136^** | 1,005 (54.8%) | 236 (12.9%) | HR: 0.9 (0.60 - 1.10)  p > 0.1^&^ | Age, sex, education, ethnicity, APOE-ε4 carrier status, smoking, cohort |
| **Raffaitin et al., 2009^137^** | NR | 208 (2.9%) | HR: 1.28 (0.92 - 1.80)  p = 0.15 | Age, gender, education, study centre |
| **Solfrizzi et al., 2010^138^** | 918 (43.8%) | 88 (4.2%) | HR: 1.00 (0.61 - 1.65)  p = NR | Age, sex, education, alcohol intake, smoking, fibrinogen, non-HDL cholesterol, ratio of apolipoprotein B to apolipoprotein A-I, coronary artery disease, stroke |
| Machado-Fragua et al., 2022^139^ | Age <60:  1,302 (17.9%)  Age 60-69:  2,111 (31.7%)  Age ≥70:  1,787 (49.5%) | Age <60:  393 (5.4%)  Age 60-69:  417 (6.3%)  Age ≥70:  264 (7.3%) | Age <60:  HR: 1.23 (0.96 - 1.57)  p = 0.098^&^  Age 60-69:  HR: 1.14 (0.91 - 1.42)  p = 0.247^&^  Age ≥70:  HR: 1.10 (0.86 - 1.40)  p = 0.495^&^ | Age, sex, education, ethnicity, birth cohort, smoking, alcohol intake, fruit and vegetable intake, physical activity |
| Qureshi et al., 2023^140^ | 73,510 (41.7%) | 5,255 (3.0%) | HR: 1.12 (1.06 - 1.18)  p < 0.0001^&^ | Age, sex, ethnicity, education, Townsend deprivation index score, household income, smoking, alcohol intake, physical activity, APOE-ε4 carrier status. |
| Qureshi et al., 2024^141^ | 7,019 (34.8%) | 2,653 (13.2%) | HR: 1.11 (1.01-1.21)  p < 0.0001^&^ | Age, sex, Townsend deprivation index, education, smoking status, alcohol intake frequency, physical activity, and APOE-ε4 carrier status |
| Ekram et al., 2023^142^ | NR | 524 (3.1%) | SHR: 0.96 (0.74 - 1.26)^$$^  p = NR | Age, gender, ethnicity, education, smoking, alcohol intake, chronic kidney disease, depression, previous cancer history |

MetS: Metabolic syndrome, NCEP-ATP III: National Cholesterol Education Program - Adult Treatment Panel III, NR: Not reported, OR: Odds Ratio, HR: Hazard Ratio, CI: Confidence Interval, HDL: High density lipoprotein, APOE: Apolipoprotein E, SHR: Sub-distribution Hazard Ratio

||: Calculated based on other available data, *: Reported for 1,936 participants in supplementary analysis, &: Additional information provided by the authors, #: among phase 2 participants (n=2,398) – chosen based on balance between longest follow-up and largest sample size, $$: based on a subset of non-frail participants in a sample of 16,965 participants.

### Table S6: Results of included studies for the association between MetS and incident Alzheimer's disease

| **Study author, publication year** | **Number and % of MetS cases** | **Number and % of incident AD** | **Effect estimate (95% CI)** | **Confounder adjustments** |
| --- | --- | --- | --- | --- |
| **Forti et al., 2010^133^** | <75 years: 133 (28.5%)  ≥75 years: 63 (22.3%) | <75 years: 18 (3.9%)  ≥75 years: 35 (12.4%) | <75 years: HR: 0.84 (0.29 - 2.44)  p = NR  ≥75 years: HR: 0.33 (0.12 - 0.94)  p = 0.04 | Age, sex, education, APOE-ε4 carrier status, physical activity, hyperhomocysteinemia, and inflammation status, history of cardiovascular disease, history of stroke |
| **Lee et al., 2020^134^** | 1,880,175 (45.8%) | ~14,509 (0.4%)^\|\|^ | HR: 1.09 (1.07 - 1.11)  p = NR | Age, sex, smoking, alcohol intake, physical activity, stroke, depression, chronic kidney disease |
| **Muller et al., 2007^136^** | 1,005 (54.8%) | 147 (8.0%) | HR: 0.90 (0.60 - 1.30)  p > 0.1^&^ | Age, sex, education, ethnicity, APOE-ε4 carrier status, smoking, cohort |
| **Raffaitin et al., 2009^137^** | NR | 134 (1.9%) | HR: 0.81 (0.50 - 1.31)  p = 0.39 | Age, gender, education, study centre |
| **Solfrizzi et al., 2010^138^** | 918 (43.8%) | 47 (2.2%) | HR: 0.83 (0.42 - 1.63)  p = NR | Age, sex, education, alcohol intake, smoking, fibrinogen, non-HDL cholesterol, ratio of apolipoprotein B to apolipoprotein A-I, coronary artery disease, stroke |

###

MetS: Metabolic syndrome, NCEP-ATP III: National Cholesterol Education Program - Adult Treatment Panel III, HR: Hazard Ratio, CI: Confidence Interval, HDL: High density lipoprotein, APOE: Apolipoprotein E, AD: Alzheimer's disease, NR: Not Reported, ||: Calculated based on other available data, &: Additional information provided by the authors.

### Table S7: Results of included studies for the association between MetS and incident vascular dementia

| **Study author, publication year** | **Number and % of MetS cases** | **Number and % of incident VaD** | **Effect estimate (95% CI)** | **Confounder adjustments** |
| --- | --- | --- | --- | --- |
| **Forti et al., 2010^133^** | <75 years: 133 (28.5%)  ≥75 years: 63 (22.3%) | <75 years: 14 (3.0%)  ≥75 years: 15 (5.3%) | <75 years: HR: 0.79 (0.23 - 2.79)  ≥75 years: HR: 1.54 (0.42 - 5.69)  p = 0.51 | Age, sex, education, APOE-ε4 carrier status, physical activity, hyperhomocysteinemia, and inflammation status, history of cardiovascular disease, history of stroke |
| **Lee et al., 2020^134^** | 1,880,175 (45.8%) | ~2,475 (0.06%)^\|\|^ | HR: 1.27 (1.22 - 1.32)  p = NR | Age, sex, smoking, alcohol intake, physical activity, stroke, depression, chronic kidney disease |
| **Muller et al., 2007^136^** | 1,005 (54.8%) | 73 (4.0%) | HR: 0.80 (0.50 - 1.30)  p > 0.1^&^ | Age, sex, education, ethnicity, APOE-ε4 carrier status, smoking, cohort |
| **Raffaitin et al., 2009^137^** | NR | 40 (0.6%) | HR: 2.42 (1.24 - 4.73)  p = 0.01 | Age, gender, education, study centre |
| **Solfrizzi et al., 2010^138^** | 918 (43.8%) | 25 (1.2%) | HR: 3.71 (1.40 - 9.83)  p = NR | Age, sex, education, alcohol intake, smoking, fibrinogen, non-HDL cholesterol, ratio of apolipoprotein B to apolipoprotein A-I, coronary artery disease, stroke |

MetS: Metabolic syndrome, NCEP-ATP III: National Cholesterol Education Program - Adult Treatment Panel III, HR: Hazard Ratio, CI: Confidence Interval, HDL: High density lipoprotein, APOE: Apolipoprotein E, VaD: Vascular dementia, NR: Not Reported.

||: Calculated based on other available data, &: Additional information provided by the authors.

### Table S8: Results of included studies for the association between MetS and other dementias

| **Study author, publication year** | **Number and % of MetS cases** | **Number and % of incident dementia** | **Effect estimate (95% CI)** | **Confounder adjustments** |
| --- | --- | --- | --- | --- |
| **Peng et al., 2018^143^** | 204 (25.9%) | 105 (13.3%) | Parkinson's Disease Dementia:   OR: 2.12 (1.57 - 2.83)  p = 0.003 | Age, sex, education, body mass index, coronary artery disease, lacunar infarction, white matter lesions, smoking, alcohol intake, baseline UPDRS score and H&Y stage |
| **Solfrizzi et al., 2010^138^** | 918 (43.8%) | 16 (0.8%) | Other dementias^^#^:  HR: 0.23 (0.06 - 1.08)  p = NR | Age, sex, education, alcohol intake, smoking, fibrinogen, non-HDL cholesterol, ratio of apolipoprotein B to apolipoprotein A-I, coronary artery disease, stroke |
| MetS: Metabolic syndrome, NCEP-ATP III: National Cholesterol Education Program - Adult Treatment Panel III, OR: Odds Ratio, HR: Hazard Ratio, CI: Confidence Interval, UPDRS: Unified Parkinson's Disease Rating Scale, H & Y Stage: Hoehn and Yahr Stage, HDL: High density lipoprotein, APOE: Apolipoprotein E, VaD: Vascular dementia, ^#: Defined as dementia in other diseases classified elsewhere (e.g., dementia in Pick's disease, Creutzfeldt - Jakob disease, Huntington's disease, Parkinson's disease, human immunodeficiency virus [HIV], or other disease), NR: Not Reported | | | | |

### Figure S1: Subgroup meta-analysis according to age group (restricted to studies of participants aged 65+ years)

**Abbreviations:** CI = Confidence Interval; df = Degrees of Freedom

**Note:** Pooled estimates were calculated using random-effects meta-analysis models

### Figure S2: Subgroup meta-analysis according to metabolic syndrome criteria used in studies – Studies using the NCEP-ATP III criteria

**Abbreviations:** CI = Confidence Interval; df = Degrees of Freedom

**Note:** Pooled estimates were calculated using random-effects meta-analysis models

### Figure S3: Subgroup meta-analysis according to metabolic syndrome criteria used in studies for all-cause dementia – Studies using the 2009 Harmonized Criteria

**Abbreviations:** CI = Confidence Interval; df = Degrees of Freedom

**Note:** Pooled estimates were calculated using random-effects meta-analysis models

### Figure S4: Subgroup meta-analysis according to ethnicity – Predominantly White populations

**Abbreviations:** CI = Confidence Interval; df = Degrees of Freedom

**Note:** Pooled estimates were calculated using random-effects meta-analysis models

### Figure S5: Subgroup meta-analysis according to ethnicity – Predominantly Non-White populations

**Abbreviations:** CI = Confidence Interval; df = Degrees of Freedom

**Note:** Pooled estimates were calculated using random-effects meta-analysis models

### Figure S6: Subgroup meta-analysis according to study quality – Excluding weak studies

**Abbreviations:** CI = Confidence Interval; df = Degrees of Freedom

**Note:** Pooled estimates were calculated using random-effects meta-analysis models

### Figure S7: Meta-analysis excluding studies without clinical adjudication of dementia diagnosis

###

**Abbreviations:** CI = Confidence Interval; df = Degrees of Freedom

**Note:** Pooled estimates were calculated using random-effects meta-analysis models

### Figure S8: Meta-analysis excluding studies that adjusted for intermediate outcomes (stroke or cardiovascular disease) in the association between MetS and vascular dementia

**Abbreviations:** CI = Confidence Interval; df = Degrees of Freedom

**Note:** Pooled estimates were calculated using random-effects meta-analysis models

### Figure S9: Meta-analysis excluding US studies (i.e., Muller, 2007)

**Abbreviations:** CI = Confidence Interval; df = Degrees of Freedom

**Note:** Pooled estimates were calculated using random-effects meta-analysis models

### Figure S10: Meta-analysis of studies investigating the association of MetS trajectories and risk of incident all-cause dementia

**Abbreviations:** CI = Confidence Interval; df = Degrees of Freedom

**Note:** Pooled estimates were calculated using random-effects meta-analysis models

### Figure S11: Meta-analysis of studies investigating the association between the (categorical) number of MetS components present and risk of all-cause dementia

**Abbreviations:** CI = Confidence Interval; df = Degrees of Freedom

**Note:** Pooled estimates were calculated using random-effects meta-analysis models

### Figure S12: Funnel plot of studies investigating the association of MetS and risk of incident A) All-cause dementia; B) Alzheimer’s disease; and C) Vascular dementia

Precision = Standard Error; Effect Sizes = Hazard Ratios

### Table S9: Egger’s test results for publication bias in meta-analyses of all-cause dementia

| **Outcome** | **t-value** | **Degrees of freedom** | **p-value** | **Bias estimate** | **Standard error** | **Tau²** |
| --- | --- | --- | --- | --- | --- | --- |
| **All-cause dementia** | -1.97 | 9 | 0.0798 | -0.56 | 0.2838 | 0.691 |

### Supplementary references

1. Alberti K, Eckel RH, Grundy SM, et al. Harmonizing the metabolic syndrome: a joint interim statement of the international diabetes federation task force on epidemiology and prevention; national heart, lung, and blood institute; American heart association; world heart federation; international atherosclerosis society; and international association for the study of obesity. *Circulation*. 2009;120(16):1640-1645.

2. Alberti K, Zimmet P, Shaw J. The metabolic syndrome—a new worldwide definition. *The Lancet*. 2005;366(9491):1059-1062.

3. Grundy SM, Brewer Jr HB, Cleeman JI, Smith Jr SC, Lenfant C. Definition of metabolic syndrome: report of the National Heart, Lung, and Blood Institute/American Heart Association conference on scientific issues related to definition. *Circulation*. 2004;109(3):433-438.

4. Einhorn D. American College of Endocrinology position statement on the insulin resistance syndrome. *Endocrine Practice*. 2003;9:5-21.

5. Grundy SM, Cleeman JI, Daniels SR, et al. Diagnosis and management of the metabolic syndrome: an American Heart Association/National Heart, Lung, and Blood Institute scientific statement. *Circulation*. 2005;112(17):2735-2752.

6. Expert Panel on Detection Evaluation and Treatment of High Blood Cholesterol in Adults. Executive summary of the third report of the National Cholesterol Education Program (NCEP) expert panel on detection, evaluation, and treatment of high blood cholesterol in adults (adult treatment panel III). *JAMA*. 2001;285(19):2486-2497.

7. Balkau B. Comment on the provisional report from the WHO consultation. European Group for the Study of Insulin Resistance (EGIR). *Diabetic Medicine*. 1999;16:442-443.

8. Alberti K, Zimmet PZ. Definition, diagnosis and classification of diabetes mellitus and its complications. Part 1: diagnosis and classification of diabetes mellitus. Provisional report of a WHO consultation. *Diabetic Medicine*. 1998;15(7):539-553.

9. Kolovou GD, Anagnostopoulou KK, Salpea KD, Mikhailidis DP. The prevalence of metabolic syndrome in various populations. *The American Journal of the Medical Sciences*. 2007;333(6):362-371.

10. Ko GT. Metabolic syndrome or “central obesity syndrome”? *Diabetes Care*. 2006;29(3):752-752.

11. Wiedman D. Globalizing the Chronicities of Modernity. *Chronic Conditions, Fluid States: Chronicity and the Anthropology of Illness Studies in Medical Anthropology*. 2010:38-53.

12. Sasya M, Devi K, Babu JK, Balaguru Rayappan JB, Krishnan UM. Metabolic Syndrome—An emerging constellation of risk factors: electrochemical detection strategies. *Sensors*. 2020;20(1):103.

13. Bray GA. *The metabolic syndrome and obesity*. Springer Science & Business Media; 2008.

14. Craiu E, Cojocaru L, Rusali A, Maxim R, Parepa I. *Dysmetabolic syndrome*. IntechOpen; 2012.

15. Hjermann I. The metabolic cardiovascular syndrome: syndrome X, Reaven's syndrome, insulin resistance syndrome, atherothrombogenic syndrome. *Journal of Cardiovascular Pharmacology*. 1992;20:S5-10.

16. Leslie BR. Metabolic syndrome: Historical perspectives. *The American Journal of the Medical Sciences*. 2005;330(6):264-268.

17. Gupta A, Gupta V. Metabolic syndrome: what are the risks for humans? *Bioscience Trends*. 2010;4(5)

18. Sarafidis PA, Nilsson PM. The metabolic syndrome: a glance at its history. *Journal of Hypertension*. 2006;24(4):621-626.

19. Han TS, Lean ME. Metabolic syndrome. *Medicine*. 2015;43(2):80-87.

20. Samson SL, Garber AJ. Metabolic syndrome. *Endocrinology and Metabolism Clinics*. 2014;43(1):1-23.

21. Balkau B, Valensi P, Eschwège E, Slama G. A review of the metabolic syndrome. *Diabetes & Metabolism*. 2007;33(6):405-413.

22. Yamaoka K, Tango T. Effects of lifestyle modification on metabolic syndrome: a systematic review and meta-analysis. *BMC Medicine*. Nov 2012;10138. doi:10.1186/1741-7015-10-138

23. Xu YH, Shen ST, Sun LZ, Yang HW, Jin B, Cao XH. Metabolic Syndrome Risk after Gestational Diabetes: A Systematic Review and Meta-Analysis. *PLoS One*. Jan 2014;9(1)e87863. doi:10.1371/journal.pone.0087863

24. Xi B, He D, Zhang M, Xue J, Zhou DH. Short sleep duration predicts risk of metabolic syndrome: A systematic review and meta-analysis. *Sleep Medicine Reviews*. Aug 2014;18(4):293-297. doi:10.1016/j.smrv.2013.06.001

25. Wong Y, Cook P, Roderick P, Somani BK. Metabolic Syndrome and Kidney Stone Disease: A Systematic Review of Literature. *Journal of Endourology*. Mar 2016;30(3):246-253. doi:10.1089/end.2015.0567

26. Wens I, Dalgas U, Stenager E, Eijnde BO. Risk factors related to cardiovascular diseases and the metabolic syndrome in multiple sclerosis - a systematic review. *Multiple Sclerosis Journal*. Oct 2013;19(12):1556-1564. doi:10.1177/1352458513504252

27. Vidigal FD, Bressan J, Babio N, Salas-Salvado J. Prevalence of metabolic syndrome in Brazilian adults: a systematic review. *BMC Public Health*. Dec 2013;131198. doi:10.1186/1471-2458-13-1198

28. Vancampfort D, Stubbs B, Mitchell AJ, et al. Risk of metabolic syndrome and its components in people with schizophrenia and related psychotic disorders, bipolar disorder and major depressive disorder: a systematic review and meta-analysis. *World Psychiatry*. Oct 2015;14(3):339-347. doi:10.1002/wps.20252

29. Thomas G, Sehgal AR, Kashyap SR, Srinivas TR, Kirwan JP, Navaneethan SD. Metabolic Syndrome and Kidney Disease: A Systematic Review and Meta-analysis. *Clinical Journal of the American Society of Nephrology*. Oct 2011;6(10):2364-2373. doi:10.2215/cjn.02180311

30. Srikanthan K, Feyh A, Visweshwar H, Shapiro JI, Sodhi K. Systematic Review of Metabolic Syndrome Biomarkers: A Panel for Early Detection, Management, and Risk Stratification in the West Virginian Population. *International Journal of Medical Sciences*. 2016;13(1):25-38. doi:10.7150/ijms.13800

31. Rosenbaum S, Stubbs B, Ward PB, Steel Z, Lederman O, Vancampfort D. The prevalence and risk of metabolic syndrome and its components among people with posttraumatic stress disorder: a systematic review and meta-analysis. *Metabolism*. Aug 2015;64(8):926-933. doi:10.1016/j.metabol.2015.04.009

32. Rodriguez-Monforte M, Sanchez E, Barrio F, Costa B, Flores-Mateo G. Metabolic syndrome and dietary patterns: a systematic review and meta-analysis of observational studies. *European Journal of Nutrition*. Apr 2017;56(3):925-947. doi:10.1007/s00394-016-1305-y

33. Ranasinghe P, Mathangasinghe Y, Jayawardena R, Hills AP, Misra A. Prevalence and trends of metabolic syndrome among adults in the asia-pacific region: a systematic review. *BMC Public Health*. Jan 2017;17101. doi:10.1186/s12889-017-4041-1

34. Rabkin SW. The Relationship Between Epicardial Fat and Indices of Obesity and the Metabolic Syndrome: A Systematic Review and Meta-Analysis. *Metabolic Syndrome and Related Disorders*. Feb 2014;12(1):31-42. doi:10.1089/met.2013.0107

35. Povel CM, Boer JMA, Reiling E, Feskens EJM. Genetic variants and the metabolic syndrome: a systematic review. *Obesity Reviews*. Nov 2011;12(11):952-967. doi:10.1111/j.1467-789X.2011.00907.x

36. Parkinson JRC, Hyde MJ, Gale C, Santhakumaran S, Modi N. Preterm Birth and the Metabolic Syndrome in Adult Life: A Systematic Review and Meta-analysis. *Pediatrics*. Apr 2013;131(4):E1240-E1263. doi:10.1542/peds.2012-2177

37. Pan A, Keum N, Okereke OI, et al. Bidirectional Association Between Depression and Metabolic Syndrome A systematic review and meta-analysis of epidemiological studies. *Diabetes Care*. May 2012;35(5):1171-1180. doi:10.2337/dc11-2055

38. Ostman C, Smart NA, Morcos D, Duller A, Ridley W, Jewiss D. The effect of exercise training on clinical outcomes in patients with the metabolic syndrome: a systematic review and meta-analysis. *Cardiovascular Diabetology*. Aug 2017;16110. doi:10.1186/s12933-017-0590-y

39. Osborn DPJ, Wright CA, Levy G, King MB, Deo R, Nazareth I. Relative risk of diabetes, dyslipidaemia, hypertension and the metabolic syndrome in people with severe mental illnesses: Systematic review and metaanalysis. *BMC Psychiatry*. Sep 2008;884. doi:10.1186/1471-244x-8-84

40. Nibali L, Tatarakis N, Needleman I, et al. Association Between Metabolic Syndrome and Periodontitis: A Systematic Review and Meta-analysis. *The Journal of Clinical Endocrinology & Metabolism*. Mar 2013;98(3):913-920. doi:10.1210/jc.2012-3552

41. Mottillo S, Filion KB, Genest J, et al. The Metabolic Syndrome and Cardiovascular Risk A Systematic Review and Meta-Analysis. *Journal of the American College of Cardiology*. Sep 2010;56(14):1113-1132. doi:10.1016/j.jacc.2010.05.034

42. Moran LJ, Misso ML, Wild RA, Norman RJ. Impaired glucose tolerance, type 2 diabetes and metabolic syndrome in polycystic ovary syndrome: a systematic review and meta-analysis. *Human Reproduction Update*. Jul-Aug 2010;16(4):347-363. doi:10.1093/humupd/dmq001

43. Mitchell AJ, Vancampfort D, Sweers K, van Winkel R, Yu WP, De Hert M. Prevalence of Metabolic Syndrome and Metabolic Abnormalities in Schizophrenia and Related Disorders-A Systematic Review and Meta-Analysis. *Schizophrenia Bulletin*. Mar 2013;39(2):306-318. doi:10.1093/schbul/sbr148

44. Mejia SB, Kendall CWC, Viguiliouk E, et al. Effect of tree nuts on metabolic syndrome criteria: a systematic review and meta-analysis of randomised controlled trials. *BMJ Open*. 2014;4(7)e004660. doi:10.1136/bmjopen-2013-004660

45. Marventano S, Salomone F, Godos J, et al. Coffee and tea consumption in relation with non-alcoholic fatty liver and metabolic syndrome: A systematic review and meta-analysis of observational studies. *Clinical Nutrition*. Dec 2016;35(6):1269-1281. doi:10.1016/j.clnu.2016.03.012

46. Marquez-Sandoval F, Macedo-Ojeda G, Viramontes-Horner D, Ballart JDF, Salvado JS, Vizmanos B. The prevalence of metabolic syndrome in Latin America: a systematic review. *Public Health Nutrition*. Oct 2011;14(10):1702-1713. doi:10.1017/s1368980010003320

47. Markopoulou P, Papanikolaou E, Analytis A, Zoumakis E, Siahanidou T. Preterm Birth as a Risk Factor for Metabolic Syndrome and Cardiovascular Disease in Adult Life: A Systematic Review and Meta-Analysis. *Journal of Pediatrics*. Jul 2019;210:69-80. doi:10.1016/j.jpeds.2019.02.041

48. Manheimer EW, van Zuuren EJ, Fedorowicz Z, Pijl H. Paleolithic nutrition for metabolic syndrome: systematic review and meta-analysis. *The American Journal of Clinical Nutrition*. Oct 2015;102(4):922-932. doi:10.3945/ajcn.115.113613

49. Mabry RM, Reeves MM, Eakin EG, Owen N. Gender differences in prevalence of the metabolic syndrome in Gulf Cooperation Council Countries: a systematic review. *Diabetic Medicine*. May 2010;27(5):593-597. doi:10.1111/j.1464-5491.2010.02998.x

50. Lloyd LJ, Langley-Evans SC, McMullen S. Childhood obesity and risk of the adult metabolic syndrome: a systematic review. *International Journal of Obesity*. Jan 2012;36(1):1-11. doi:10.1038/ijo.2011.186

51. Lipovec NC, Beijers R, van den Borst B, Doehner W, Lainscak M, Schols A. The Prevalence of Metabolic Syndrome In Chronic Obstructive Pulmonary Disease: A Systematic Review. *Journal of Chronic Obstructive Pulmonary Disease*. 2016;13(3):399-406. doi:10.3109/15412555.2016.1140732

52. Kelishadi R, Mansourian M, Heidari-Beni M. Association of fructose consumption and components of metabolic syndrome in human studies: A systematic review and meta-analysis. *Nutrition*. May 2014;30(5):503-510. doi:10.1016/j.nut.2013.08.014

53. Grossmann M, Hoermann R, Wittert G, Yeap BB. Effects of testosterone treatment on glucose metabolism and symptoms in men with type 2 diabetes and the metabolic syndrome: a systematic review and meta-analysis of randomized controlled clinical trials. *Clinical Endocrinology*. Sep 2015;83(3):344-351. doi:10.1111/cen.12664

54. Gami AS, Witt BJ, Howard DE, et al. Metabolic syndrome and risk of incident cardiovascular events and death - A systematic review and meta-analysis of longitudinal studies. *Journal of the American College of Cardiology*. Jan 2007;49(4):403-414. doi:10.1016/j.jacc.2006.09.032

55. Gacci M, Corona G, Vignozzi L, et al. Metabolic syndrome and benign prostatic enlargement: a systematic review and meta-analysis. *BJU International*. Jan 2015;115(1):24-31. doi:10.1111/bju.12728

56. Friend A, Craig L, Turner S. The Prevalence of Metabolic Syndrome in Children: A Systematic Review of the Literature. *Metabolic Syndrome and Related Disorders*. Apr 2013;11(2):71-80. doi:10.1089/met.2012.0122

57. Esposito K, Kastorini CM, Panagiotakos DB, Giugliano D. Mediterranean diet and metabolic syndrome: An updated systematic review. *Reviews in Endocrine & Metabolic Disorders*. Sep 2013;14(3):255-263. doi:10.1007/s11154-013-9253-9

58. Esposito K, Chiodini P, Colao A, Lenzi A, Giugliano D. Metabolic Syndrome and Risk of Cancer A systematic review and meta-analysis. *Diabetes Care*. Nov 2012;35(11):2402-2411. doi:10.2337/dc12-0336

59. Esposito K, Chiodini P, Capuano A, et al. Colorectal cancer association with metabolic syndrome and its components: a systematic review with meta-analysis. *Endocrine*. Dec 2013;44(3):634-647. doi:10.1007/s12020-013-9939-5

60. Esposito K, Chiodini P, Capuano A, et al. Metabolic syndrome and postmenopausal breast cancer: systematic review and meta-analysis. *Journal of the North American Menopause Society*. Dec 2013;20(12):1301-1309. doi:10.1097/gme.0b013e31828ce95d

61. Dunkley AJ, Charles K, Gray LJ, Camosso-Stefinovic J, Davies MJ, Khunti K. Effectiveness of interventions for reducing diabetes and cardiovascular disease risk in people with metabolic syndrome: systematic review and mixed treatment comparison meta-analysis. *Diabetes Obesity & Metabolism*. Jul 2012;14(7):616-625. doi:10.1111/j.1463-1326.2012.01571.x

62. Chu P, Gotink RA, Yeh GY, Goldie SJ, Hunink MGM. The effectiveness of yoga in modifying risk factors for cardiovascular disease and metabolic syndrome: A systematic review and meta-analysis of randomized controlled trials. *European Journal of Preventive Cardiology*. Feb 2016;23(3):291-307. doi:10.1177/2047487314562741

63. Canuto R, Garcez AS, Olinto MTA. Metabolic syndrome and shift work: A systematic review. *Sleep Medicine Reviews*. Dec 2013;17(6):425-431. doi:10.1016/j.smrv.2012.10.004

64. Brand JS, van der Tweel I, Grobbee DE, Emmelot-Vonk MH, van der Schouw YT. Testosterone, sex hormone-binding globulin and the metabolic syndrome: a systematic review and meta-analysis of observational studies. *International Journal of Epidemiology*. Feb 2011;40(1):189-207. doi:10.1093/ije/dyq158

65. Bora E, Akdede BB, Alptekin K. The relationship between cognitive impairment in schizophrenia and metabolic syndrome: a systematic review and meta-analysis. *Psychological Medicine*. Apr 2017;47(6):1030-1040. doi:10.1017/s0033291716003366

66. Bergmann N, Gyntelberg F, Faber J. The appraisal of chronic stress and the development of the metabolic syndrome: a systematic review of prospective cohort studies. *Endocrine Connections*. Jun 2014;3(2):R55-R80. doi:10.1530/ec-14-0031

67. Bassi N, Karagodin I, Wang S, et al. Lifestyle Modification for Metabolic Syndrome: A Systematic Review. *American Journal of Medicine*. Dec 2014;127(12)1242.e1. doi:10.1016/j.amjmed.2014.06.035

68. Baranova A, Tran TP, Birerdinc A, Younossi ZM. Systematic review: association of polycystic ovary syndrome with metabolic syndrome and non-alcoholic fatty liver disease. *Alimentary Pharmacology & Therapeutics*. Apr 2011;33(7):801-814. doi:10.1111/j.1365-2036.2011.04579.x

69. Ballestri S, Zona S, Targher G, et al. Nonalcoholic fatty liver disease is associated with an almost twofold increased risk of incident type 2 diabetes and metabolic syndrome. Evidence from a systematic review and meta-analysis. *Journal of Gastroenterology and Hepatology*. May 2016;31(5):936-944. doi:10.1111/jgh.13264

70. Armstrong AW, Harskamp CT, Armstrong EJ. Psoriasis and metabolic syndrome: A systematic review and meta-analysis of observational studies. *Journal of the American Academy of Dermatology*. Apr 2013;68(4):654-662. doi:10.1016/j.jaad.2012.08.015

71. Amiot MJ, Riva C, Vinet A. Effects of dietary polyphenols on metabolic syndrome features in humans: a systematic review. *Obesity Reviews*. Jul 2016;17(7):573-586. doi:10.1111/obr.12409

72. Arvanitakis Z, Shah RC, Bennett DA. Diagnosis and management of dementia. *JAMA*. 2019;322(16):1589-1599.

73. Burns A, Iliffe S. Clinical review: dementia. *BMJ*. 2009;338:b75.

74. Livingston G, Huntley J, Sommerlad A, et al. Dementia prevention, intervention, and care: 2020 report of the Lancet Commission. *The Lancet*. 2020;396(10248):413-446.

75. Prince M, Bryce R, Albanese E, Wimo A, Ribeiro W, Ferri CP. The global prevalence of dementia: a systematic review and metaanalysis. *Alzheimer's & Dementia*. 2013;9(1):63-75. e2.

76. AlDawsari A, Bushell TJ, Abutheraa N, Sakata S, Al Hussain S, Kurdi A. Use of sedative-hypnotic medications and risk of dementia: A systematic review and meta-analysis. *British Journal of Clinical Pharmacology*. doi:10.1111/bcp.15113

77. Biessels GJ, Staekenborg S, Brunner E, Brayne C, Scheltens P. Risk of dementia in diabetes mellitus: a systematic review. *Lancet Neurology*. Jan 2006;5(1):64-74. doi:10.1016/s1474-4422(05)70284-2

78. Bos D, Wolters FJ, Darweesh SKL, et al. Cerebral small vessel disease and the risk of dementia: A systematic review and meta-analysis of population-based evidence. *Alzheimers & Dementia*. Nov 2018;14(11):1482-1492. doi:10.1016/j.jalz.2018.04.007

79. Bougea A, Anagnostouli M, Angelopoulou E, Spanou I, Chrousos G. Psychosocial and Trauma-Related Stress and Risk of Dementia: A Meta-Analytic Systematic Review of Longitudinal Studies. *Journal of Geriatric Psychiatry and Neurology*. Jan 2022;35(1):24-37. 0891988720973759. doi:10.1177/0891988720973759

80. Bucci T, Menichelli D, Pignatelli P, Triggiani M, Violi F, Pastori D. Relationship of Antiphospholipid Antibodies to Risk of Dementia: A Systematic Review. *Journal of Alzheimer’s Disease*. 2019;69(2):561-576. doi:10.3233/jad-181294

81. Campbell JM, Stephenson MD, de Courten B, Chapman I, Bellman SM, Aromataris E. Metformin Use Associated with Reduced Risk of Dementia in Patients with Diabetes: A Systematic Review and Meta-Analysis. *Journal of Alzheimer’s Disease*. 2018;65(4):1225-1236. doi:10.3233/jad-180263

82. Cao L, Tan L, Wang HF, et al. Dietary Patterns and Risk of Dementia: a Systematic Review and Meta-Analysis of Cohort Studies. *Molecular Neurobiology*. Nov 2016;53(9):6144-6154. doi:10.1007/s12035-015-9516-4

83. Charoenngam N, Rittiphairoj T, Ponvilawan B, Ungprasert P. Patients with psoriasis have a higher risk of dementia: A systematic review and meta-analysis. *Indian Journal of Dermatology Venereology & Leprology*. May-Jun 2021;87(3):364-370. Pmid 33666046. doi:10.25259/ijdvl_732_19

84. Chu CS, Tseng PT, Stubbs B, et al. Use of statins and the risk of dementia and mild cognitive impairment: A systematic review and meta-analysis. *Scientific Reports*. Apr 2018;85804. doi:10.1038/s41598-018-24248-8

85. Cui HY, Wang Y, Li F, et al. Quantifying observational evidence for risk of dementia following androgen deprivation therapy for prostate cancer: an updated systematic review and meta-analysis. *Prostate Cancer and Prostatic Diseases*. Mar 2021;24(1):15-23. doi:10.1038/s41391-020-00267-3

86. de Almondes KM, Costa MV, Malloy-Diniz LF, Diniz BS. Insomnia and risk of dementia in older adults: Systematic review and meta-analysis. *Journal of Psychiatric Research*. Jun 2016;77:109-115. doi:10.1016/j.jpsychires.2016.02.021

87. Della Gatta F, Lacorte E, Fabrizi E, et al. Exploring the association of early life physical activity and risk of dementia: a systematic review. *Minerva Medica*. Aug 2021;112(4):448-455. doi:10.23736/s0026-4806.21.07542-x

88. den Brok M, van Dalen JW, Abdulrahman H, et al. Antihypertensive Medication Classes and the Risk of Dementia: A Systematic Review and Network Meta-Analysis. *Journal of the American Medical Directors Association*. Jul 2021;22(7):1386-+. doi:10.1016/j.jamda.2020.12.019

89. Desai M, Nutalapati V, Srinivasan S, et al. Proton pump inhibitors do not increase the risk of dementia: a systematic review and meta-analysis of prospective studies. *Diseases of the Esophagus*. Oct 2020;33(10)doaa041. doi:10.1093/dote/doaa041

90. Desai R, John A, Stott J, Charlesworth G. Living alone and risk of dementia: A systematic review and meta-analysis. *Ageing Research Reviews*. Sep 2020;62101122. doi:10.1016/j.arr.2020.101122

91. Diniz BS, Teixeira AL, Cao F, et al. History of Bipolar Disorder and the Risk of Dementia: A Systematic Review and Meta-Analysis. *The American Journal of Geriatric Psychiatry*. Apr 2017;25(4):357-362. doi:10.1016/j.jagp.2016.11.014

92. Fan L, Xu WH, Cai YL, Hu YX, Wu CK. Sleep Duration and the Risk of Dementia: A Systematic Review and Meta-analysis of Prospective Cohort Studies. *Journal of the American Medical Directors Association*. Dec 2019;20(12):1480-+. doi:10.1016/j.jamda.2019.06.009

93. Gates NJ, Sachdev PS, Singh MAF, Valenzuela M. Cognitive and memory training in adults at risk of dementia: A Systematic Review. *BMC Geriatrics*. 2011;1155. doi:10.1186/1471-2318-11-55

94. Ge YJ, Xu W, Tan CC, Tan L. Blood-based biomarkers in hypothalamic-pituitary axes for the risk of dementia or cognitive decline: a systematic review and meta-analysis. *Aging US*. Oct 2020;12(20):20350-20365. doi:10.18632/aging.103813

95. Georgakis MK, Beskou-Kontou T, Theodoridis I, Skalkidou A, Petridou ET. Surgical menopause in association with cognitive function and risk of dementia: A systematic review and meta-analysis. *Psychoneuroendocrinology*. Aug 2019;106:9-19. doi:10.1016/j.psyneuen.2019.03.013

96. Hou XH, Feng L, Zhang C, Cao XP, Tan L, Yu JT. Models for predicting risk of dementia: a systematic review. *Journal of Neurology Neurosurgery and Psychiatry*. Apr 2019;90(4):373-379. doi:10.1136/jnnp-2018-318212

97. Islam MM, Iqbal U, Walther B, et al. Benzodiazepine Use and Risk of Dementia in the Elderly Population: A Systematic Review and Meta-Analysis. *Neuroepidemiology*. 2016;47(3-4):181-191. doi:10.1159/000454881

98. Kuiper JS, Zuidersma M, Voshaar RCO, et al. Social relationships and risk of dementia: A systematic review and meta-analysis of longitudinal cohort studies. *Ageing Research Reviews*. Jul 2015;22:39-57. doi:10.1016/j.arr.2015.04.006

99. Kuring JK, Mathias JL, Ward L. Risk of Dementia in persons who have previously experienced clinically-significant Depression, Anxiety, or PTSD: A Systematic Review and Meta-Analysis. *Journal of Affective Disorders*. Sep 2020;274:247-261. doi:10.1016/j.jad.2020.05.020

100. Lazzari C, Rabottini M. COVID-19, loneliness, social isolation and risk of dementia in older people: a systematic review and meta-analysis of the relevant literature. *International Journal of Psychiatry in Clinical Practice*. doi:10.1080/13651501.2021.1959616

101. Lee J. Influence of Cardiorespiratory Fitness on Risk of Dementia and Dementia Mortality: A Systematic Review and Meta-Analysis of Prospective Cohort Studies. *Journal of Aging and Physical Activity*. Oct 2021;29(5):878-885. doi:10.1123/japa.2019-0493

102. Lee ZX, Ang E, Lim XT, Arain SJ. Association of Risk of Dementia With Direct Oral Anticoagulants Versus Warfarin Use in Patients With Non-valvular Atrial Fibrillation: A Systematic Review and Meta-analysis. *Journal of Cardiovascular Pharmacology*. Jan 2021;77(1):22-31. doi:10.1097/fjc.0000000000000925

103. Li M, Luo Z, Yu SS, Tang ZY. Proton pump inhibitor use and risk of dementia Systematic review and meta-analysis. *Medicine*. Feb 2019;98(7)e14422. doi:10.1097/md.0000000000014422

104. Mongkhon P, Naser AY, Fanning L, et al. Oral anticoagulants and risk of dementia: A systematic review and meta analysis of observational studies and randomized controlled trials. *Neuroscience and Biobehavioral Reviews*. Jan 2019;96:1-9. doi:10.1016/j.neubiorev.2018.10.025

105. Muzambi R, Bhaskaran K, Brayne C, Davidson JA, Smeeth L, Warren-Gash C. Common Bacterial Infections and Risk of Dementia or Cognitive Decline: A Systematic Review. *Journal of Alzheimer’s Disease*. 2020;76(4):1609-1626. doi:10.3233/jad-200303

106. Nelson ME, Jester DJ, Petkus AJ, Andel R. Cognitive Reserve, Alzheimer's Neuropathology, and Risk of Dementia: A Systematic Review and Meta-Analysis. *Neuropsychology Review*. Jun 2021;31(2):233-250. doi:10.1007/s11065-021-09478-4

107. Opie RS, Ralston RA, Walker KZ. Adherence to a Mediterranean-style diet can slow the rate of cognitive decline and decrease the risk of dementia: a systematic review. *Nutrition & Dietetics*. Sep 2013;70(3):206-217. doi:10.1111/1747-0080.12016

108. Penninkilampi R, Casey AN, Singh MF, Brodaty H. The Association between Social Engagement, Loneliness, and Risk of Dementia: A Systematic Review and Meta-Analysis. *Journal of Alzheimer’s Disease*. 2018;66(4):1619-1633. doi:10.3233/jad-180439

109. Penninkilampi R, Eslick GD. A Systematic Review and Meta-Analysis of the Risk of Dementia Associated with Benzodiazepine Use, After Controlling for Protopathic Bias. *CNS Drugs*. Jun 2018;32(6):485-497. doi:10.1007/s40263-018-0535-3

110. Peters R, Booth A, Rockwood K, Peters J, D'Este C, Anstey K. Combining modifiable risk factors and risk of dementia: a systematic review and meta-analysis. *BMJ Open*. Jun 2019;9(1)e022846. doi:10.1136/bmjopen-2018-022846

111. Ponvilawan B, Charoenngam N, Rittiphairoj T, Ungprasert P. Ankylosing spondylitis is associated with an increased risk of dementia: A systematic review and meta-analysis. *International Journal of Rheumatic Diseases*. Nov 2020;23(11):1452-1459. doi:10.1111/1756-185x.13920

112. Shi L, Chen SJ, Ma MY, et al. Sleep disturbances increase the risk of dementia: A systematic review and meta-analysis. *Sleep Medicine Reviews*. Aug 2018;40:4-16. doi:10.1016/j.smrv.2017.06.010

113. Sommerlad A, Ruegger J, Singh-Manoux A, Lewis G, Livingston G. Marriage and risk of dementia: systematic review and meta-analysis of observational studies. *Journal of Neurology Neurosurgery and Psychiatry*. Mar 2018;89(3):231-238. doi:10.1136/jnnp-2017-316274

114. Tan EYL, Kohler S, Hamel REG, Munoz-Sanchez JL, Verhey FRJ, Ramakers I. Depressive Symptoms in Mild Cognitive Impairment and the Risk of Dementia: A Systematic Review and Comparative Meta-Analysis of Clinical and Community-Based Studies. *Journal of Alzheimer’s Disease*. 2019;67(4):1319-1329. doi:10.3233/jad-180513

115. Tian XL, Guo XK, Xia XS, Yu HB, Li X, Jiang AL. The comparison of cognitive function and risk of dementia in CKD patients under peritoneal dialysis and hemodialysis: A PRISMA-compliant systematic review and meta-analysis. *Medicine*. Feb 2019;98(6)e14390. doi:10.1097/md.0000000000014390

116. Ungprasert P, Wijarnpreecha K, Thongprayoon C. Rheumatoid arthritis and the risk of dementia: A systematic review and meta-analysis. *Neurology India*. Jan-Feb 2016;64(1):56-61. doi:10.4103/0028-3886.173623

117. Velosa J, Delgado A, Finger E, Berk M, Kapczinski F, Cardoso TD. Risk of dementia in bipolar disorder and the interplay of lithium: a systematic review and meta-analyses. *Acta Psychiatrica Scandinavica*. Jun 2020;141(6):510-521. doi:10.1111/acps.13153

118. Wang J, Xu WH, Sun SS, Yu SY, Fan L. Headache disorder and the risk of dementia: a systematic review and meta-analysis of cohort studies. *Journal of Headache and Pain*. Oct 2018;1995. doi:10.1186/s10194-018-0925-4

119. Weave J, Bennett EE, Ranker L, et al. Exposure to Air Pollution in Relation to Risk of Dementia and Related Outcomes: An Updated Systematic Review of the Epidemiological Literature. *Environmental Health Perspectives*. Sep 2021;129(9)096001. doi:10.1289/ehp8716

120. Wolters FJ, Segufa RA, Darweesh SKL, et al. Coronary heart disease, heart failure, and the risk of dementia: A systematic review and meta-analysis. *Alzheimers & Dementia*. Nov 2018;14(11):1493-1504. doi:10.1016/j.jalz.2018.01.007

121. Yan SJ, Fu WN, Wang C, et al. Association between sedentary behavior and the risk of dementia: a systematic review and meta-analysis. *Translational Psychiatry*. Apr 2020;10(1)112. doi:10.1038/s41398-020-0799-5

122. Zheng YB, Shi L, Zhu XM, et al. Anticholinergic drugs and the risk of dementia: A systematic review and meta-analysis. *Neuroscience and Biobehavioral Reviews*. Aug 2021;127:296-306. doi:10.1016/j.neubiorev.2021.04.031

123. Zhou ZK, Liang YF, Zhang XQ, et al. Fibrinogen and risk of dementia: A systematic review and meta-analysis. *Neuroscience and Biobehavioral Reviews*. May 2020;112:353-360. doi:10.1016/j.neubiorev.2020.02.022

124. Zhou ZK, Zhong SS, Liang YF, et al. Serum Uric Acid and the Risk of Dementia: A Systematic Review and Meta-Analysis. *Frontiers in Aging Neuroscience*. Feb 2021;13625690. doi:10.3389/fnagi.2021.625690

125. Zuin M, Roncon L, Passaro A, Bosi C, Cervellati C, Zuliani G. Risk of dementia in patients with atrial fibrillation: Short versus long follow-up. A systematic review and meta-analysis. *International Journal of Geriatric Psychiatry*. Oct 2021;36(10):1488-1500. doi:10.1002/gps.5582

126. Kuźma E, Littlejohns TJ, Khawaja AP, Llewellyn DJ, Ukoumunne OC, Thiem U. Visual impairment, eye diseases, and dementia risk: a systematic review and meta-analysis. *Journal of Alzheimer's Disease*. 2021;83(3):1073-1087.

127. Scottish Intercollegiate Guidelines Network. Search filters. <https://www.sign.ac.uk/what-we-do/methodology/search-filters/>

128. Thomas B, Ciliska D, Dobbins M, Micucci S. A process for systematically reviewing the literature: providing the research evidence for public health nursing interventions. *Worldviews on Evidence‐Based Nursing*. 2004;1(3):176-184.

129. Higgins JP, Thomas J, Chandler J, et al. *Cochrane handbook for systematic reviews of interventions*. John Wiley & Sons; 2019.

130. Creavin ST, Gallacher J, Bayer A, Fish M, Ebrahim S, Ben-Shlomo Y. Metabolic syndrome, diabetes, poor cognition, and dementia in the Caerphilly prospective study. *Journal of Alzheimer's Disease*. 2012;28(4):931-939.

131. Exalto LG, Van Der Flier WM, Van Boheemen CJ, et al. The metabolic syndrome in a memory clinic population: relation with clinical profile and prognosis. *Journal of the Neurological Sciences*. 2015;351(1-2):18-23.

132. Fan YC, Chou CC, You SL, Sun CA, Chen CJ, Bai CH. Impact of worsened metabolic syndrome on the risk of dementia: a nationwide cohort study. *Journal of the American Heart Association*. 2017;6(9):e004749.

133. Forti P, Pisacane N, Rietti E, et al. Metabolic syndrome and risk of dementia in older adults. *Journal of the American Geriatrics Society*. 2010;58(3):487-492.

134. Lee JE, Shin DW, Han K, et al. Changes in metabolic syndrome status and risk of dementia. *Journal of Clinical Medicine*. 2020;9(1):122.

135. Ng TP, Feng L, Nyunt MSZ, et al. Metabolic syndrome and the risk of mild cognitive impairment and progression to dementia: follow-up of the Singapore longitudinal ageing study cohort. *JAMA Neurology*. 2016;73(4):456-463.

136. Muller M, Tang M-X, Schupf N, Manly JJ, Mayeux R, Luchsinger JA. Metabolic syndrome and dementia risk in a multiethnic elderly cohort. *Dementia and Geriatric Cognitive Disorders*. 2007;24(3):185-192.

137. Raffaitin C, Gin H, Empana J-P, et al. Metabolic syndrome and risk for incident Alzheimer's disease or vascular dementia: the Three-City Study. *Diabetes Care*. 2009;32(1):169-174.

138. Solfrizzi V, Scafato E, Capurso C, et al. Metabolic syndrome and the risk of vascular dementia: the Italian Longitudinal Study on Ageing. *Journal of Neurology, Neurosurgery & Psychiatry*. 2010;81(4):433-440.

139. Machado-Fragua MD, Fayosse A, Yerramalla MS, et al. Association of metabolic syndrome with incident dementia: role of number and age at measurement of components in a 28-year follow-up of the Whitehall II cohort study. *Diabetes Care*. 2022;45(9):2127-2135.

140. Qureshi D, Collister J, Allen NE, Kuźma E, Littlejohns T. Association between metabolic syndrome and risk of incident dementia in UK Biobank. *Alzheimer's & Dementia*. 2023;

141. Qureshi D, Luben R, Hayat S, et al. Role of age and exposure duration in the

association between metabolic syndrome and risk of incident dementia: a prospective

cohort study. *The Lancet Healthy Longevity*. 2024;

142. Ekram A, Espinoza S, Ernst M, et al. The Association between Metabolic Syndrome, Frailty and disability-free survival in Healthy Community-dwelling older adults. *The Journal of Nutrition, Health and Aging*. 2023;27(1):1-9.

143. Peng Z, Dong S, Tao Y, et al. Metabolic syndrome contributes to cognitive impairment in patients with Parkinson's disease. *Parkinsonism & Related Disorders*. 2018;55:68-74.
